# Combinatorial Subset Difference—IoT-Friendly Subset Representation and Broadcast Encryption

**DOI:** 10.3390/s20113140

**Published:** 2020-06-02

**Authors:** Jiwon Lee, Seunghwa Lee, Jihye Kim, Hyunok Oh

**Affiliations:** 1 Department of Information System, Hanyang University, Seoul 04763, Korea; jiwonlee@hanyang.ac.kr; 2 Department of Security Enhanced Smart Electric Vehicle, Kookmin University, Seoul 02707, Korea; ttyhgo@kookmin.ac.kr; 3 Department of Electrical Engineering, Kookmin University, Seoul 02707, Korea; jihyek@kookmin.ac.kr

**Keywords:** broadcast encryption, public key encryption, IP multicast, subset difference, wildcard

## Abstract

In the Internet of Things (IoT) systems, it is often required to deliver a secure message to a group of devices. The public key broadcast encryption is an efficient primitive to handle IoT broadcasts, by allowing a user (or a device) to broadcast encrypted messages to a group of legitimate devices. This paper proposes an IoT-friendly subset representation called Combinatorial Subset Difference (CSD), which generalizes the existing subset difference (SD) method by allowing wildcards (*) in any position of the bitstring. Based on the CSD representation, we first propose an algorithm to construct the CSD subset, and a CSD-based public key broadcast encryption scheme. By providing the most general subset representation, the proposed CSD-based construction achieves a minimal header size among the existing broadcast encryption. The experimental result shows that our CSD saves the header size by 17% on average and more than 1000 times when assuming a specific IoT example of IP address with 20 wildcards and $2^{20}$ total users, compared to the SD-based broadcast encryption. We prove the semantic security of CSD-based broadcast encryption under the standard *l*-BDHE assumption, and extend the construction to a chosen-ciphertext-attack (CCA)-secure version.

## 1. Introduction

In the recent applications, the Internet of Things (IoT) systems are more likely to involve multicast of privacy-sensitive information; for example, an IoT sensor network in the smart city involves personal whereabouts transmitted to multiple devices, and an IoT medical system requires sensitive health information to be delivered to the authorized devices. In these IoT secure communications, cryptographic primitives can provide useful functionalities and efficiencies. Especially, advanced encryption protocols like attribute-based encryption or broadcast encryption can handle simultaneous multicast efficiently; attribute-based encryption is often applied to the IoT devices [1,2], and broadcast encryption, which is a specific and efficient version of the attribute-based encryption, is also considered [3] for secure firmware updates in IoT.

Until now, existing cryptographic applications in IoT systems such as those in References [1,2,3,4] assumed device ID as an intrinsic identity string. However, in a network environment in which the IoT device is identified by an IP address, it is useful to use IP address as an ID. Moreover, an arbitrary network group can be effectively represented by IP address including wildcard (*), and if a part of IP is connected to attributes, a device group specified by a set of attributes can also be effectively expressed. This flexible representation denoted by IP characteristics or attributes covers any specific group even without knowing every individual ID predefined. This paper focuses on the efficient and scalable public key broadcast encryption scheme that transmits a message securely to any group of legitimate receiver represented as an flexible IP address in the IoT environment.

**Broadcast Encryption.** The public key broadcast encryption is an effective cryptosystem for a secure group communication which allows a user (or a device) to broadcast secure messages so that only privileged users (or devices) can decrypt them. To minimize the broadcasting payload, the design of broadcast encryption adopts the hybrid encryption; an original message is often encrypted with a simple symmetric key encryption (e.g., advanced encryption standard (AES)), and the applied symmetric key becomes an official message for public key broadcast encryption. The encrypted key is called a header, which is often regarded as an official ciphertext in the broadcast encryption literature. For the transmission, the privileged users are organized within multiple subsets according to the subset representation. Then the broadcast encryption algorithm is repeatedly applied to each subset; the headers are collected into a vector so that the receiver can find and decrypt the header for his own subset. The symmetric key encryption of the original message is broadcast to the entire users, but only privileged users can decrypt the symmetric key from the header and obtain the message.

**Subset Representation.** In the broadcast encryption, a subset representation is an important factor which strictly determines the number of subsets. The number of subsets decides the total header size since each subset requires an individual header. Therefore, it is recommended to maintain the subset representation as general as possible so that the representation can cover as many as privileged users, which can decrease the number of subsets. In the broadcast encryption, the subset representation is considered separately: the construction assumes there already exists pre-defined subsets covering the given privileged users.

**Figure 1:**
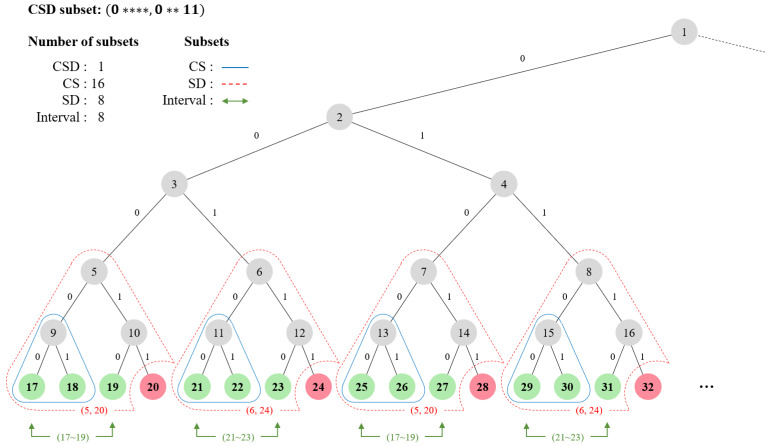
Example: subset representations in existing methods and Combinatorial Subset Difference (CSD).

**Existing Methods.** The well-known subset representations are complete subtree (CS), subset difference (SD), and interval. They are all based on the binary tree structure, where the users are denoted as leaf nodes of a binary tree. Figure 1 visualizes each representation with an example where green nodes are privileged and red nodes are revoked.

The CS representation covers the privileged users by denoting the parent node; each parent node itself is a subset which includes the descendant leaf nodes. For the example in Figure 1, node 9 covers users 17 and 18, and node 19 covers only user 19. The CS representation requires 8 subsets to cover the privileged users in Figure 1.

The SD representation extends the CS by enhancing expressiveness; its covers users by denoting the difference of two subtrees as (c,d) where *c* is a covered set and *d* is a revoked set. For the example in Figure 1, a subset (5,20) which subtracts subtree 20 from subtree 5 covers users 17, 18, and 19. Since it generalizes the CS representation by adding a revoked set expression, the SD representation requires 4 subsets to cover the privileged users which is less than the CS representation.

The interval representation covers users within an interval, by denoting the left and right edge of the interval. For the example in Figure 1, a subset (17∼19) includes users 17, 18, and 19. The interval representation requires 4 subsets to cover the privileged users, which is comparable to the SD  representation.

**Limitations.** The main restriction of the existing methods is that they are all limited to the *hierarchical* tree structure. When expressing the node with a binary label instead of a number, tree-based representations cannot effectively handle non-hierarchical combinations required to represent flexible IP addresses we consider. That is, as in Figure 1, each node can be expressed with a bitstring label which consists of {0,1,*}, by considering the left branch as 0, right branch as 1, and both as wildcard (*) starting from the root. For instance, node 8 can be expressed as a label 011, and a label 01* can include nodes 7 (010) and 8 (011). In this case, the limitation is that each bit is determined from the root to the leaf node following the tree hierarchy; the wildcard cannot exist before 0 or 1 since the parent should be determined before the child nodes. For example, a label *00 is not expressible since the wildcard stands before 0, which makes it as a non-existent node in a binary tree.

A main concern is that this limitation prevents the broadcast encryption from being efficiently applied to the IoT systems. Consider the secure group communication of IoT systems where a device is identified by its IP address combined with attributes which is not restricted to the hierarchy. In other words, the wildcard occurs in any position to express a specific group representation  (e.g., 0***.**1.***.001). However, since existing representation methods limit the position of wildcards as we described above, they cannot cover the general and flexible IP addresses within a single subset, which leads to a large number of subsets.

**Combinatorial Subset Difference.** To overcome the hierarchical limitations, we propose a new IoT-friendly subset representation called *combinatorial subset difference (CSD)*, which extends the SD representation by allowing any combinatorial labels, that is, allowing wildcards in any position. Specifically, the CSD expresses a subset similar to SD as (c,d), but where *c* is a covered label and *d* is a revoked label that can allow wildcards in any bits. Our CSD is the most general representation among the existing methods, indicating that any subset expressed in the existing representation can be transformed to the CSD subset. Therefore, the CSD can achieve minimal number of subsets in any cases. To cover the privileged users in the example of Figure 1 (by expressing each user as a label), the CSD requires only a single subset (0****,0**11) while SD and interval representations require 4 different subsets.

To adopt the CSD representation in practical broadcast encryption, it is required to construct an algorithm that can output appropriate CSD subsets from the given input of privileged users. Thus, we propose a heuristic algorithm which generates CSD subsets from the list of users. We first construct an SD algorithm that can output SD subsets from the input of privileged users, then extend the algorithm to cover the general CSD cases. The proposed CSD algorithm generates *r* subsets for the worst case while the SD generates 2r−1 for the worst case, where *r* is the number of revoked users. For any cases, the algorithm guarantees that the number of CSD subsets are always no more than the SD subsets.

**CSD-based broadcast encryption.** Designing the broadcast encryption is independent of the subset representation; since we proposed a new subset representation, it is also required to design a new broadcast encryption that can adopt the input of CSD subsets. In this paper, we propose a new CSD-based public key broadcast encryption that has a minimal total header size among the existing broadcast encryption due to the minimal number of subsets. The minimal header size indicates minimal transmission cost, which can be suitable for the IoT system applications. Moreover, our CSD-based broadcast encryption improves the encryption time and decryption time while maintaining the order of key sizes.

Table 1 shows the comparison between the CSD and the existing public key broadcast encryption schemes. The public key SD is from the specifically measured by applying the public key lifting transformation [5], which combines the hierarchical identity-based encryption (HIBE) [6] to the symmetric key SD-based broadcast encryption [7,8] from the advanced access content system (AACS) DVD standard [9]. The interval refers to the Lin’s construction [10], which is based on a new interval representation from binary trees. The revocable SD refers to Lee and Park’s construction [11], which is based on the same SD representation, but improves the order of key sizes and enhances identity revocation algorithm. The elements in revocable SD is within a composite order group, where the element size is 8 times larger than the normal prime order group for the security; the size of composite order group elements are denoted as ×8. The header size refers to the total header size in the worst case, which is the product of the number of group elements per subset and the number of subsets. Among the state-of-the-art public key broadcast encryption, our CSD achieves a minimal header size of 2r, due to the minimal number of subsets.

The CSD also improves the encryption time and decryption time, compared to the other constructions. Nevertheless, the CSD still maintains the public key size within O(logn) and the secret key size within $O(\log^{2}n)$, which is comparable to the existing public key broadcast encryption. To verify the theoretical improvements, we implemented the CSD, SD, and interval broadcast encryption on the Intel Edison embedded systems: the experimental results show that our CSD reduces the header size by 1000 times on average when applied to the IP address examples, with also improving both encryption and decryption time, compared to the existing methods.

The security of our CSD-based public key broadcast encryption is proven under the standard bilinear diffie-hellman exponent (l−BDHE) assumption; we first prove the semantic security (CPA-secure) of our original construction, and then extend the construction to a CCA-secure version.

**Contributions.** We now summarize the contribution of our paper, in the sense of practicality and theoretical advances.

- **CSD subset representation:** we propose a new IoT-friendly subset representation called combinatorial subset difference (CSD), the most general representation that achieves minimal number of subsets among the existing methods.
- **CSD covering algorithm:** we construct a subset generation algorithm for the proposed CSD representation, which can output CSD subsets from the given input of privileged users.
- **CSD-based broadcast encryption:** we propose a new public key broadcast encryption based on the CSD representation, which achieves a minimal header size due to the minimal number of subsets, suitable for the secure group communication in IoT systems. It also improves encryption and decryption time compared to the existing public key broadcast encryption.
- **Security proof:** we provide a formal security proof for the semantic security of our construction under the l−BDHE assumption, in the standard model.
- **CCA-secure extension:** we also propose an extended CCA-secure version of our CSD-based public key broadcast encryption, along with a formal proof for the CCA security.
- **Implementation:** we implemented our construction on the actual IoT device to verify the performance, and provide experimental results showing comparison between the CSD and the existing broadcast encryption schemes.

This paper is organized as follows. Section 2 states related works. Section 3 describes the subset construction algorithm. Section 4 organizes preliminaries for broadcast encryption systems. We present our broadcast encryption scheme in Section 5, and prove the security for security analysis in Section 6. Then we extend it to a CCA-secure broadcast encryption scheme in Section 7, and prove the security in Section 8. Section 9 shows experimental results. Section 10 makes conclusion.

## 2. Related Work

### 2.1. Secure Multicast

Secure multicast is a traditional encryption model for multi-user infrastructure, which considers an environment that encrypts messages to multiple users simultaneously. Most of the secure multicast protocols work with a central manager responsible for the broadcast, and focuses on improving the efficiency of key distribution and transmission.

Among the secure multicast research, Chu et al. [12] proposed a notable end-to-end protocol which can secure both message and the copyright of the content. Their work support the dynamic group, which can let users join/leave or expel users during the system running. However, the security of their protocol relies on informal analysis against few attacks, and the fundamental technique remains on multiple encryption which let the encryption required for the number of total users in the group.

For the efficiency, Kumar et al. [13] proposed a protocol based on the centralized key distribution. By providing more efficient key distribution from the hierarchical tree structure, their protocol could achieve more flexible group control and efficient computation. Still, the total communication relies on the individual transmissions, which leads to a considerably large transmission load.

Recently, Wang et al. [14] implemented the concept of secure multicast on the IoT environment, by proposing an end-to-end key agreement protocol for the IoT devices. It focuses on the authentication of devices when interacting with the user’s server, where the user can open a secure channel if the key is once agreed. For the security, their protocol deploy fuzzy extractor, which can use user’s biometric information such as fingerprints as a secret key.

### 2.2. Broadcast Encryption

The broadcast encryption is a traditional cryptosystem where one can encrypt a message to the group of multiple users. In the broadcast encryption, the encryption can be handled for each group rather than individuals; it is an efficient primitive to be deployed on the secure multicast.

There are many constructions for the broadcast encryption, with different functionalities and improvements [5,6,8,15,16,17,18,19,20,21,22,23,24,25,26,27,28,29]. In the cryptographic literature, the broadcast encryption is categorized by two different criteria: symmetric/public key, and stateful/stateless.

**Symmetric vs. Public.** Similar to the categorization of standard encryption systems, the broadcast encryption can be either be based on the symmetric key settings or the public key (or asymmetric) settings. If the broadcast encryption is based on the symmetric key [25,29], only a user with the key can encrypt the message, that is, only the central authority can broadcast messages to other users. On the other hand, if the broadcast encryption is based on the public key [8,10,23], anyone can broadcast messages to other users.

**Stateful vs. Stateless.** The stateful broadcast encryption and stateless broadcast encryption is determined by the key update. In the stateful broadcast encryption [28], the secret key of users can be updated time to time, in order to help the revocation (proper broadcast). However, in the stateless broadcast encryption [8,25,26], the system allows the key exchange for only once in the initial setup. Stateful broadcast encryption can be easier to design with the help of key updates, but it also involves a price of simultaneous key managements. On the other hand, in the stateless broadcast encryption, the system can change revocation without any key management after the initialization.

  In many interactive IoT systems, it is often required to allow each device to broadcast messages rather than restricting the broadcast to the central manager, which requires public key broadcast encryption. Also, stateless broadcast encryption is more appropriate than the stateful settings for the IoT systems, since small and numerous IoT devices are not suitable for frequent updates and managements. In these sense, the proposed broadcast encryption for combinatorial subset difference is a stateless public key broadcast encryption, which satisfies the IoT requirements.

## 3. Proposed Subset Construction Algorithm

Despite the efficiency of the combinatorial subset difference (CSD) representation, it is a remaining work to construct a concrete algorithm which returns CSD subsets from a list of privileged/revoked users. In this section, we propose a heuristic algorithm for the CSD subset construction. The proposed algorithm does not assure the optimal minimization of the subsets. However, it guarantees the number of subsets in CSD is no more than the existing subset difference (SD) algorithm [8]. For the worst case, it generates no more than *r* subsets while the existing SD algorithm generates 2r−1 subsets.

Since CSD is the generalization of SD, we first explain the how the SD algorithm minimizes the number of subsets. Then we show how to extend it to the CSD algorithm, by focusing on the difference between the SD and the CSD.

In the SD (and CSD) representation, a subset is represented as S=(c,d), where *c* is a covered set (nodes to include), and *d* is a revoked set (nodes to exclude). For instance, in a tree structure, a subset *S* includes all descendants of the node *c*, except all descendants of the node *d*.

**Figure 2:**
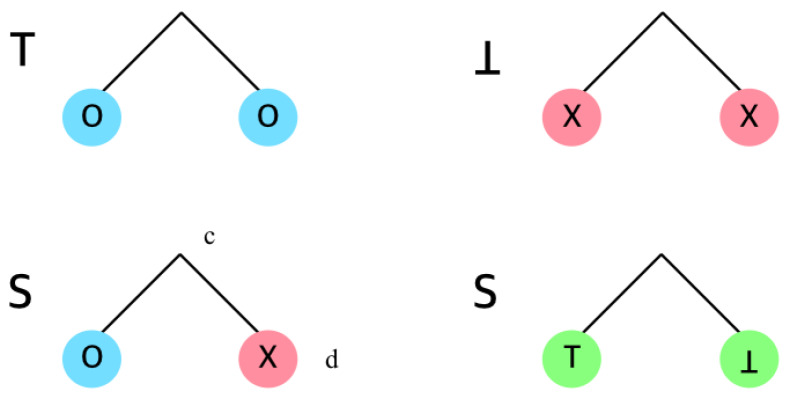
The definition of node types. Node O is a legitimate node and node X is a revoked node.

In the SD algorithm, an internal node *c* can be categorized as one of the following three types, as illustrated in Figure 2, depending on its children nodes.

- Type ⊤: There is no revoked descendant of *c*.
- Type ⊥: Node *c* is excluded by its parent, because either it is already covered by another subset or its all descendants are revoked.
- Type *S*: Node *c* becomes a subset (c,d), where all descendants of *d* are either revoked or already covered by other subsets.

**Figure 3:**
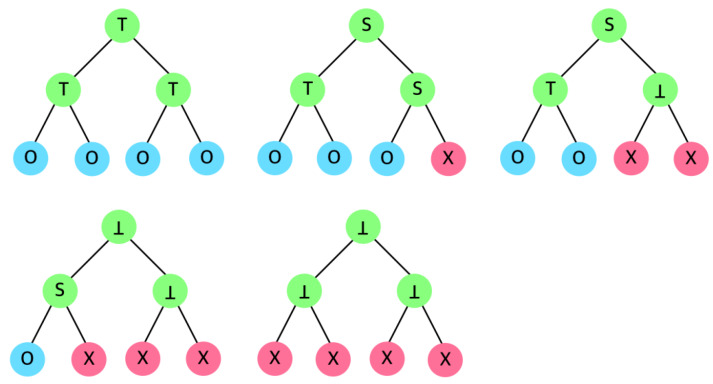
Node type examples in Subset Difference (SD) (and CSD).

The node type is determined by the types of its children. Therefore, the internal node type is determined from bottom to top, recursively. As in Figure 3, combining two internal nodes identifies the type (Figure 2) of their parent.

**Figure 4:**
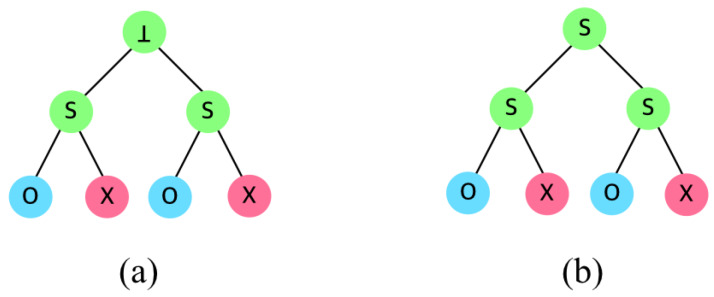
Category difference between (**a**) SD and (**b**) CSD.

For both SD and CSD, the examples in Figure 3 are valid. However, as in Figure 4, the CSD is different from SD in case when two *S* nodes are combined; the SD let the parent of two *S* be a type ⊥, while CSD let it be another *S*. In the SD, a set can be fixed only if its descendants are all ⊤ or ⊥. Therefore, two *S* nodes cannot be merged into a single set; the parent node becomes ⊥ since two *S* type nodes are determined as individual subsets in SD. However, in CSD, the parent node becomes another *S* by merging two *S* nodes, without generating two subsets.

**Figure 5:**
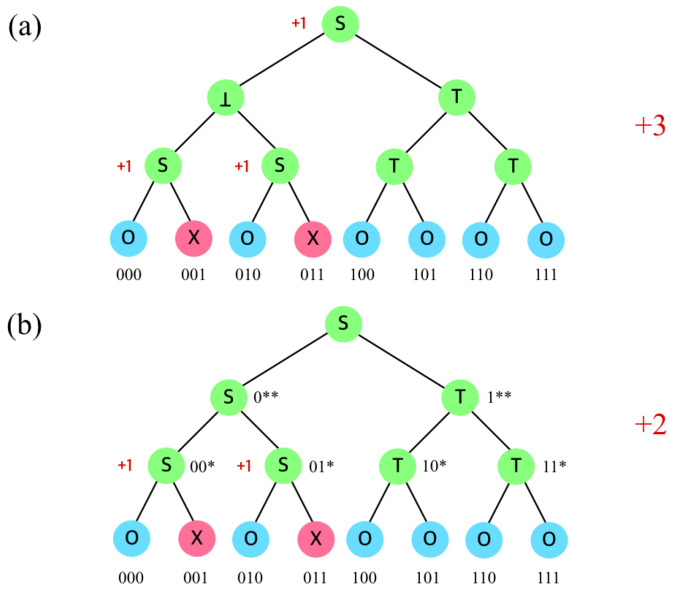
Subset construction in (**a**) SD, and (**b**) CSD.

Figure 5 shows an example where SD and CSD outputs different results, where users (nodes) 1 and 3 are revoked among 8 total users. When representing the revoked nodes in a binary format, 001 and 011 are revoked. The parent nodes are denoted as *. For instance, 00* is a parent node of 000 and 001, and 01* is a parent node of 010 and 011. The type of node 000 is ⊤ since nothing is revoked, and the type of node 001 is ⊥ since it is revoked. The type of node 00* is *S*, since its children 000 is included while its other children 001 is excluded, and it requires a subset (00*,001) to be covered. Similarly, the type of node 01* is *S*. In case of the SD algorithm, the node 0** with type *S* children cannot be an inclusion label since both of its children are labeled as two different subsets ((00*,001) and (01*,011)) which cannot be merged. Thus, 0** should be excluded which determines its type as ⊥. Then the type of the root note is *S* since its left child is ⊥ and its right child is ⊤. As a result, three subsets are generated as (***,0**), (00*,001), and (01*,011).

On the other hand, in the CSD algorithm, node 0** is treated specially, different from the SD. Although the type of its children are both *S*, it is not necessary to exclude the node; if the node can be merged with a sibling node of type ⊤, it is possible to represent the node without generating a new subset. In Figure 5, the node 0** has two subsets (00*,000) and (01*,010), and it has a sibling node 1** of type ⊤. When merging subsets (00*,000) and (01*,010) with (1**,⊥), it generates (*0*,000) and (*1*,010) where *0* and *1* can include all eight users while 000 and 010 can exclude 000 and 010 since the CSD allows * in the middle of the representation regardless of the tree hierarchy. As a result, the CSD generates two subsets as (*0*,001) and (*1*,011), which does not generates subsets more than the number of revoked users.

Table 2 describes node type resolution in SD and CSD, where $T_{0}$ is the type of left child and $T_{1}$ is the type of right child. The subset generation indicates whether a new subset generation is required for the resolved node type; a new subset should be generated when one child is included while the other child is excluded. The only difference between SD and CSD occurs when the type of both children are *S*: the node is merged into type ⊥ in SD while it is merged into another *S* type in CSD.

Algorithm 1 shows how to build subsets in both SD and CSD methods. The only difference appears at line 27: the CSD proceeds an additional line 27 while the SD does not. Consequently, as shown in Table 2, line 29 ($resolveType(T_{0},T_{1})$) returns different values.
**Algorithm 1** Subset construction for SD and CSD.1:**function**sd(Set, Unresolved, i,prefix,revSet)2: **if** i=depth **then**3:  **if** |revSet|>0 **then**4:   **return** ⊥5:  **else**6:   **return** ⊤7:  **end if**8: **else if** |revSet|=0 **then**9:  **return** ⊤10: **end if**11: $S_{0}=\left\{r|r\in revSet,bit\left(r,i+1\right)=0\right\}$12: $S_{1}=\left\{r|r\in revSet,bit\left(r,i+1\right)=1\right\}$13: $T_{0}\leftarrow$sd(Set, $U_{0}$, $i+1,prefix||0,S_{0}$)14: $T_{1}\leftarrow$sd(Set, $U_{1}$, $i+1,prefix||1,S_{1}$)15: **if** $\left(T_{0}=⊥\right)\wedge \left(T_{1}=⊤\right)$ **then**16:  s←createSubset(prefix,i,0)17:  Unresolved←s; Set←Set∪s18: **else if** $\left(T_{0}=⊤\right)\wedge \left(T_{1}=⊥\right)$ **then**19:  s←createSubset(prefix,i,1)20:  Unresolved←s; Set←Set∪s21: **else if** $\left(T_{0}=S\right)\wedge \left(T_{1}=⊤\right)$ **then**22:  $\forall \left(c,d\right)\in U_{0},c_{i}\leftarrow *$; $Unresolved\leftarrow U_{0}$23: **else if** $\left(T_{0}=⊤\right)\wedge \left(T_{1}=S\right)$ **then**24:  $\forall \left(c,d\right)\in U_{1},c_{i}\leftarrow *$; $Unresolved\leftarrow U_{1}$25: **else if** $Combinatorial\wedge \left(T_{0}=S\right)\wedge \left(T_{1}=S\right)$ **then**26:  // Only used for CSD27:  $Unresolved\leftarrow U_{0}\cup U_{1}$28: **end if**29: **return** $resolveType(T_{0},T_{1})$30:**end function**31:**procedure**main32: $revSet\leftarrow \{revoked\;user\;IDs^{\prime }\}$33: header←sd(⊥,⊥,0,⊥,revSet)34:**end procedure**

In the algorithm, the subset construction starts with an ID set of revoked users as an input. Function sd reads each ID of revoked users from the most significant bit (MSB) to the least significant bit (LSB), where *i* denotes the current index. When *i* reaches the LSB, the user is determined as either revoked or legitimate. Therefore, it returns ⊥ or ⊤ as in lines from 2 to 7. Even if *i* does not reach LSB, it returns ⊤ if there is no revoked user. In line 11 through 12, the revoked users are divided into two sets, depending on the next bit of user’s ID, and the function sd is called recursively for each set denoting tree traverse in lines 13 through 14. If the types of each children is ⊥ and ⊤, a new subset should be created as described in lines 16 and 19. Function createSubset(prefix,i,b) is for creating a subset (c,d) where *c* denotes an inclusion label and *d* denotes an exclusion label. Label *c* covers all users with $prefix_{1},\ldots ,prefix_{i},*,\ldots$, and label *d* covers all users with $prefix_{1},\ldots ,prefix_{i},b,*,\ldots$, where $prefix_{k}$ is a *k*-th bit of prefix. When the type of a child is *S* and the other one is ⊤, each inclusion label in the unresolved subset is updated to include the type ⊤ child, as in lines 22 and 24. In the CSD, the unresolved subsets for type *S* children are preserved as unresolved, since inclusion labels in unresolved subsets will be updated in case they include a type ⊤ node as in line 27.

**Theorem** **1.** *For the combinatorial subset difference (CSD) representation, Algorithm 1 generates at most r subsets for r revoked users.*

**Proof.**  As illustrated in Table 2, a subset is generated when a child node has a ⊥ type. The resolved type becomes ⊥, at least only if a child node is ⊥. A leaf node (user) has type ⊥ only if it is revoked. If there are *r* revoked nodes, there are at most *r* leaf nodes with type ⊥. Hence, the number of generated subsets is no more than *r*. □

Unlike CSD, the number of subsets in the SD can exceed *r*, since a node can still be type ⊥ even if both children have type *S*.

For the worst case comparison, it is straightforward to deduce that the number of subsets in CSD does not exceed the number of subsets in SD. For the subset generation, a subset is created when a child is ⊥ and the other child is ⊤, both in SD and CSD algorithm. Also in both algorithms, a node can be type ⊤ when its children are both ⊤, while it should be ⊥ when one child is ⊥ and the other child is ⊥ or *S*. However, in SD, a node becomes ⊥ when its children are both *S*, which may require an additional subset. Therefore, the number of subsets in CSD algorithm is no more than that of SD algorithm.

## 4. Preliminaries

### 4.1. Public Key Broadcast Encryption

We follow the standard definition of public key broadcast encryption from Reference [23]. In the broadcast encryption, it is often combined with the symmetric key encryption, where the encryption encapsulates the session key instead of arbitrary messages, and the encryption is performed for each subset *S*. Formally, a public key broadcast encryption Π contains three functions as below:

- $(PK,{\left\{SK_{ID}\right\}}_{ID\in {\left\{0,1\right\}}^{l}})\leftarrow$Setup(l,m): the setup algorithm initializes the system by setting up public parameters, and generating private keys for stateless devices. From user ID length *l* and session key length *m*, the setup returns a public key PK and $2^{l}$ secret keys ${\left\{SK_{ID}\right\}}_{ID\in {\left\{0,1\right\}}^{l}}$.
- $\left(S,Hdr,C_{M}\right)=\left(Hdr,K\right)\leftarrow$Encrypt(PK,S): the encrypt algorithm is performed for each subset *S*. It first outputs a header Hdr and a message encryption key K∈K from the input of subset $S\subseteq {\left\{0,1\right\}}^{l}$ and a public key PK. Then, for the message *M* to be broadcast to a set *S*, let $C_{M}$ be an encryption of *M* under the session key *K*. The transmission for the users in *S* is selected as $\left(S,Hdr,C_{M}\right)$, where Hdr is called broadcast ciphertext and $C_{M}$ is called broadcast body.
- K∈K←Decrypt($S,ID,SK_{ID},Hdr$): the decrypt algorithm is performed by the individual privileged user. From the input of subset $S\subseteq {\left\{0,1\right\}}^{l}$, user ID $ID\in {\left\{0,1\right\}}^{l}$, secret key $SK_{ID}$, and a header Hdr, if ID∈S (that is privileged) then it produces the message encryption key K∈K. Then the key *K* is utilized to decrypt the $C_{M}$ to retrieve plaintext *M*.

The encryption system must let every user in *S* obtain message *M* correctly. In other words, for all $S\subseteq {\left\{0,1\right\}}^{l}$ and all ID∈S, if $\left(PK,\left(SK_{ID_{0^{l}}},\ldots ,SK_{ID_{1^{l}}}\right)\right)\overset{\$}{\leftarrow }\mathrm{Setup}\left(l,m\right)$, $\left(Hdr,K\right)\overset{\$}{\leftarrow }\mathrm{Encrypt}\left(\mathrm{PK},S\right)$ then it should satisfy $\mathrm{Decrypt}(S,\mathrm{ID},{\mathrm{SK}}_{\mathrm{ID}},\mathrm{Hdr})=K$.

For the security definition of the broadcast encryption, we describe the selective security for chosen plaintext attacks (IND-sID-CPA-security) and chosen ciphertext attacks (IND-sID-CCA-security) as in Reference [23]. Then we separate the security notions for single-set security and multi-set security, depending on the number of representations of the challenged sets. Finally we show that the single-set security implies the multi-set security.

### 4.2. Security Definition

We define the single-set security by the selective game between the challenger C and the adversary A. Both C and A knows the ID length *l* and the session key length *m* as a default inputs. The security game proceed as follows:

Init: A begins by selecting a set $S^{*}$ to claim the users which it intends to attack.
Setup: C runs Setup(l,m) to get a public key PK and secret keys $SK_{0^{l}},\ldots ,SK_{1^{l}}$, and gives A the PK and all secret keys $SK_{ID}$ for $ID\notin \;S^{*}$.
Query phase1: *(optional for CCA)*A can adaptively issue decryption queries $q_{1},\ldots ,q_{m}$; each query consists of (ID,S,Hdr) for $S\subseteq S^{*}$ and ID∈S. C replies the query by $\mathrm{Decrypt}(S,\mathrm{ID},{\mathrm{SK}}_{\mathrm{ID}},\mathrm{Hdr})$.
Challenge: C runs $\mathrm{Encrypt}(S^{*},\mathrm{PK})$ to get $\left(Hdr^{*},K\right)$ for K∈K. Then, C flips a coin b∈{0,1}. If b=1, C sets $K^{*}=K$, otherwise C chooses a random R∈K and sets $K^{*}=R$ to respond ($Hdr^{*},K^{*}$) back to A.
Query phase2: *(optional for CCA)*A can continue asking decryption queries $q_{m+1}$, ⋯, $q_{q_{D}}$; each query consists of (ID,S,Hdr) for $S\subseteq S^{*}$ and ID∈S, but the only constraint is $Hdr\neq Hdr^{*}$. C replies the query same as in phase 1.
Guess: A guesses $b^{\prime }\in \left\{0,1\right\}$ for *b*. If $b=b^{\prime }$, A wins the game.

Let ${\mathrm{AdvSSBr}}_{A,\Pi }\left(l,m\right)$ be the advantage of A to win the game above.

**Definition** **1.** *A public key broadcast encryption *Π* for a single-set is (t,ϵ,l,m)-CPA secure (or $\left(t,\epsilon ,l,m,q_{D}\right)$-CCA secure), if for every t-time adversary $A^{\prime }$ (for CCA: which makes at most $q_{D}$ decryption queries) we have $|{\mathrm{AdvSSBr}}_{A,\Pi }\left(l,m\right)-1/2|<$ϵ.*

A multi-set security is also defined by the selective game between the Ch and A, similar to the single-set security game above, but the only difference is that the challenged set is represented by multiple subsets.

Init: $A^{\prime }$ begins by selecting a set $S^{*}=\left(S_{1}^{*},\ldots ,S_{w}^{*}\right)$ to claim users which it intends to attack.
Setup: C runs Setup(l,m) to get a public key PK and secret keys $SK_{0^{l}},\ldots ,SK_{1^{l}}$, and gives $A^{\prime }$ the PK and all secret keys $SK_{ID}$ for $ID\notin \;S^{*}$.
Query phase1: *(optional for CCA)*$A^{\prime }$ can adaptively issue decryption queries $q_{1},\ldots ,q_{d}$; each query consists of (ID,S,Hdr) where $S\subseteq S_{i}^{*}$ for all *i* and ID∈S. C replies the query by $\mathrm{Decrypt}(S,\mathrm{ID},{\mathrm{SK}}_{\mathrm{ID}},\mathrm{Hdr})$.
Challenge: C runs $\mathrm{Encrypt}(S_{i}^{*},\mathrm{PK})$ for i=1,…,w to get $\left(Hdr_{i}^{*},K_{i}\right)$ for K∈K. Then, C flips a coin b∈{0,1}. If b=1, C sets $K^{*}=\left(K_{1},\ldots ,K_{w}\right)$, otherwise C chooses randoms $R_{i}\in K$ for i=1,…,w and sets $K^{*}=\left(R_{1},\ldots ,R_{w}\right)$ to respond ($Hdr^{*},K^{*}$) back to $A^{\prime }$.
Query phase2: *(optional for CCA)*$A^{\prime }$ can continues asking decryption queries $q_{q_{d}+1}$, ⋯, $q_{q_{D}}$; each query consists of (ID,S,Hdr) with $S\subseteq S_{i}^{*}$ for all *i* and ID∈S, but the only constraint is $Hdr\neq Hdr^{*}$. C replies the query same as in phase 1.
Guess: A guesses $b^{\prime }\in \left\{0,1\right\}$ for *b*. If $b=b^{\prime }$, A wins the game.

Let ${\mathrm{AdvMSBr}}_{A^{\prime },\Pi }\left(l,m\right)$ be the advantage of $A^{\prime }$ to win the above game.

**Definition** **2.** *A public key broadcast encryption $\Pi^{\prime }$ for multi-set is $\left(t,\epsilon^{\prime },l,m\right)$-CPA secure (or $\left(t,\epsilon^{\prime },l,m,q_{D}\right)$-CCA secure), if for every t-time adversary $A^{\prime }$ (for CCA: which makes at most $q_{D}$ decryption queries) we have $|{\mathrm{AdvMSBr}}_{A^{\prime },\Pi^{\prime }}\left(l,m\right)-1/2|<$ϵ.*

Finally, we show the single-set security implies the multi-set security.

**Theorem** **2.** Suppose a single-set public key broadcast encryption *Π* is $\left(t,\epsilon ,l,m,q_{D}\right)$-CCA secure. Then a multi-set public key broadcast encryption $\Pi^{\prime }$ is $\left(t,\epsilon^{\prime },l,m,q_{D}\right)$-CCA secure for arbitrary $\epsilon^{\prime }<\epsilon *w$, where w is the number of subsets [30].

**Proof.**  For the proof sketch, the basic idea is to convert the challenge key in the multi-set scheme from a real key set $K^{*}$ to a random key set ${\bar{K}}^{*}$ by using hybrid games which change each key in the single-set scheme from a real key to a random key. If the adversary cannot distinguish the changes of each key in the single-set scheme, then it also cannot distinguish the changes of challenge key in the multi-set scheme since the number of hybrid games is within polynomial. Suppose that a challenge set is given as $S^{*}=\left(S_{1},S_{2},\ldots ,S_{w}\right)$ for polynomial *w* and the corresponding key set is described as $K^{*}=\left(K_{1},\ldots ,K_{w}\right)$ s.t. $K_{i}$ is the key for $S_{i}$. The hybrid games $G_{0},\ldots ,G_{h},\ldots ,G_{w}$ for the proof are defined as follows:

**Game** $G_{0}$ In this game, all keys $K_{j}$ are real key from an encryption on the set $S_{j}$. That is, the challenge key $K^{*}$ is a set of real keys.
**Game** $G_{h}$ This game is almost identical to the game $G_{h-1}$ except the key $K_{h}$ since $K_{h}$ in this game is a random key. Specifically, in this game, the key $K_{j}$ for j≤h is a random key and the key $K_{j}$ for h<j is a real key.
**Game** $G_{w}$ In this game, all keys $K_{j}$ are random keys. That is, the challenge key $K^{*}$ is a set of random keys $\bar{K^{*}}$.

Let $S_{A}^{G_{h}}$ be the event that A outputs 1 in $G_{h}$. A distinguishes $G_{h-1}$ from $G_{h}$ by the advantage of the single-set security. Thus, we have that

$$\begin{array}{cc} Pr\left[S_{A}^{G_{0}}\right]-Pr\left[S_{A}^{G_{w}}\right]=Pr\left[S_{A}^{G_{0}}\right]+\sum_{h=1}^{w-1}(Pr\left[S_{A}^{G_{h}}\right] & -Pr\left[S_{A}^{G_{h}}\right])-Pr\left[S_{A}^{G_{w}}\right]\\ \; & \leq \sum_{h=1}^{w}\left|Pr\left[S_{A}^{G_{h-1}}\right]-Pr\left[S_{A}^{G_{h}}\right]\right|\leq 2w\;\cdot \;{\mathrm{AdvSSBr}}_{A}\left(˘\right). \end{array}$$

Finally, we obtain the inequality relation as follows:

$$\begin{array}{cc} {\mathrm{AdvMSBr}}_{A^{\prime }}\left(˘\right) & =|Pr\left[b=1\right]\;\cdot \;Pr\left[b=b^{\prime }|b=1\right]+Pr\left[b=0\right]\;\cdot \;Pr\left[b=b^{\prime }|b=0\right]-\frac{1}{2}|\\ \; & =\;|\frac{1}{2}\;\cdot \;Pr\left[b^{\prime }=1|b=1\right]+\frac{1}{2}\;\cdot \;\left(1-Pr\left[b^{\prime }=1|b=0\right]\right)-\frac{1}{2}]|\\ \; & =\frac{1}{2}\;\cdot \;\left|Pr\left[b^{\prime }=1|b=1\right]-Pr\left[b^{\prime }=1|b=0\right]\right|\\ \; & \leq \frac{1}{2}\;\cdot \;\left|Pr\left[S_{A}^{G_{0}}\right]-Pr\left[S_{A}^{G_{w}}\right]\right|\leq w\;\cdot \;{\mathrm{AdvSSBr}}_{A}\left(˘\right). \end{array}$$

 □

The above game can be transformed to define semantic security for a public key broadcast encryption system if the attacker is not allowed to issue decryption queries.

**Definition** **3.** *A public key broadcast encryption system is (t,ϵ,l,m) semantically secure if it is (t,ϵ,l,m,0)-CCA secure.*

For the scheme construction, we first provide a semantically-secure scheme, and then extend it to gain CCA security.

### 4.3. Bilinear Groups and Pairings

In References [31,32], the bilinear maps and bilinear map groups are defined as follows:

- There exists two multiplicative groups G and $G_{1}$, which are cyclic groups of prime order *p*.
- Let *g* be a generator of the group G.

For two groups G and $G_{1}$ as above, the bilinear map is defined as a map $e:G\times G\to G_{1}$, which satisfies the following properties:

- The map is bilinear: for all u,v∈G and a,b∈Z, $e\left(u^{a},v^{b}\right)=e{\left(u,v\right)}^{ab}$
- The map is non-degenerate: e(g,g)≠1.

G is a bilinear group if the operation in G is efficient and there is a group $G_{1}$ with an efficiently computable bilinear map $e:G\times G\to G_{1}$.

### 4.4. Computational Complexity Assumptions

The security of our scheme is based on the bilinear Diffie-Hellman Exponent (BDHE) assumption which is commonly used as in References [6,23]. It is a natural extension of bilinear Diffie-Hellman Inversion (BDHI) assumption.

Let G be bilinear group of prime order *p*. The *l*-BDHE problem in G are stated as follows: given a vector of 2l+1 elements

$$\left(h,g,g^{\alpha },g^{\left(\alpha^{2}\right)},\ldots ,g^{\left(\alpha^{l}\right)},g^{\left(\alpha^{l+2}\right)},\ldots ,g^{\left(\alpha^{2l}\right)}\right)\in G^{2l+1}$$

as input, output $e{\left(h,g\right)}^{\alpha^{l+1}}\in G_{1}$. For simplicity, after *g* and α are determined, we use $y_{i}$ to denote $y_{i}=g^{\alpha^{i}}\in G$. An algorithm A has advantage ϵ in solving *l*-BDHE in G if

$$Pr[A\left(h,g,y_{1},\ldots ,y_{l},y_{l+2},\ldots ,y_{2l}\right)=e\left(h,y_{l+1}\right)]\geq \epsilon ,$$

where the probability is over the random choices g,h∈G, α in $Z_{p}$, and A’s random bits. The decisional version of the *l*-BDHE problem is defined in a similar manner; let ${\vec{y}}_{g,\alpha ,l}=\left(y_{1},\ldots ,y_{l},y_{l+2},\ldots ,y_{2l}\right)$. An algorithm B which outputs b∈{0,1} has advantage ϵ in solving decisional *l*-BDHE if

$$\begin{array}{c} |Pr\left[B\left(h,g,{\vec{y}}_{g,\alpha ,l},e\left(\hat{g},y_{l+1}\right)\right)=0\right]-Pr\left[B\left(h,g,{\vec{y}}_{g,\alpha ,l},T\right)=0\right]|\geq \epsilon , \end{array}$$

where the probability is over the random choices of g,h∈G, α in $Z_{p}$, $T\in G_{1}$, and B’s random bits.

**Definition** **4.** *The (decisional) (t,ϵ,l)-BDHE assumption holds in G, if for any t-time adversary the advantage is less than ϵ on solving the (decisional) l-BDHE problem in G.*

The *t* and ϵ are often omitted, and referred as (decisional) *l*-BDHE in G.

## 5. CSD-based Broadcast Encryption

In this section, we present our public key broadcast encryption based on the combinatorial subset difference (CSD) representation. The CSD-based broadcast encryption accepts an input of CSD subset *S*; as standard broadcast encryption, it runs the broadcast encryption algorithm for each subset *S*. We first show the formal construction in Section 5.1, along with appropriate examples to aid the comprehension. Then we provide the formal proof for the collusion-resistant semantic security in Section 6.

### 5.1. Main Scheme

The main formal construction of the CSD-based public key broadcast encryption for a CSD subset *S* is described as follows:

Setup(*l*,*m*): The maximum number of users are set as $2^{l}$, which can be interpreted as *l*-level public key broadcast encryption, and the message space is set as ${\left\{0,1\right\}}^{m}$. To initialize and generate the keys, the setup algorithm selects random integers $\alpha ,\beta \in Z_{p}$, and O(l) random group elements g∈G, $g_{2},g_{3},h_{1,0},h_{1,1},\ldots ,h_{l,0}$, $h_{l,1},k_{1,0},k_{1,1},\ldots ,k_{l,0},k_{l,1}\in G$. Then it computes $g_{1}=g^{\alpha }\in G$. The public key is set as

$$\begin{array}{c} PK\leftarrow (\hat{g},\hat{g_{1}},g,g_{2},g_{3},h_{1,0},h_{1,1},\ldots ,h_{l,0},h_{l,1},k_{1,0},k_{1,1},\ldots ,k_{l,0},k_{l,1}). \end{array}$$

A master secret key is set as $master-key=g_{2}^{\alpha }$.

For an identity $ID=b_{1}\cdots b_{l}$, the setup algorithm generates a secret key $SK_{ID}$ from the master secret key. It selects random $r_{1},\ldots ,r_{l}\in Z_{p}$ and set $SK_{ID}=\left(SK_{ID,1},\ldots ,SK_{ID,l}\right)$ as

(1) $$\begin{array}{cc} SK_{ID,i}=({\hat{g}}^{r_{i}},g_{2}^{\alpha } & {\left(h_{1,b_{1}}\ldots h_{l,b_{l}}k_{i,{\bar{b}}_{i}}g_{3}\right)}^{r_{i}},\\ \; & h_{1,{\bar{b}}_{1}}^{r_{i}},\ldots ,h_{l,{\bar{b}}_{l}}^{r_{i}},k_{1,0}^{r_{i}},k_{1,1}^{r_{i}},\ldots ,k_{i-1,0}^{r_{i}},k_{i-1,1}^{r_{i}},k_{i,b_{i}}^{r_{i}},k_{i+1,0}^{r_{i}},k_{i+1,1}^{r_{i}},\ldots ,k_{l,0}^{r_{i}},k_{l,1}^{r_{i}})\in G^{3l+1}, \end{array}$$

where ${\bar{b}}_{i}$ represents a bit *NOT* of $b_{i}$, that is, $1-b_{i}$.

**Example** **1.** *For an identity ID=010,*

$$\begin{array}{cc} SK_{010}= & (g^{r_{1}},g_{2}^{\alpha }{\left(h_{1,0}h_{2,1}h_{3,0}k_{1,1}g_{3}\right)}^{r_{1}},h_{1,1}^{r_{1}},h_{2,0}^{r_{1}},h_{3,1}^{r_{1}},k_{1,0}^{r_{1}},k_{2,0}^{r_{1}},k_{2,1}^{r_{1}},k_{3,0}^{r_{1}},k_{3,1}^{r_{1}},\\ \; & g^{r_{2}},g_{2}^{\alpha }{\left(h_{1,0}h_{2,1}h_{3,0}k_{2,0}g_{3}\right)}^{r_{2}},h_{1,1}^{r_{2}},h_{2,0}^{r_{2}},h_{3,1}^{r_{2}},k_{1,0}^{r_{2}},k_{1,1}^{r_{2}},k_{2,1}^{r_{2}},k_{3,0}^{r_{2}},k_{3,1}^{r_{2}},\\ \; & g^{r_{3}},g_{2}^{\alpha }{\left(h_{1,0}h_{2,1}h_{3,0}k_{3,1}g_{3}\right)}^{r_{3}},h_{1,1}^{r_{3}},h_{2,0}^{r_{3}},h_{3,1}^{r_{3}},k_{1,0}^{r_{3}},k_{1,1}^{r_{3}},k_{2,0}^{r_{3}},k_{2,1}^{r_{3}},k_{3,0}^{r_{3}}). \end{array}$$

Encrypt(PK,S): The encrypt algorithm takes a subset corresponding to the combinatorial subset difference (CSD) representation as an input. The CSD subset is represented as S=(c,d), for $c,d\in {\left\{0,1,*\right\}}^{l}$. The label c determines the covered (included) nodes, and the label d determines the revoked (excluded) nodes. The labels can express wildcard (*), which can embrace multiple nodes. For example, a subset (**0*,0*01) indicates all users **0* except users in 0*01. **0* includes 0000,0001,0100,0101,1000,1001,1100,1101 and 0*01 includes 0001,0101, therefore, label (**0*,0*01) covers 0000,0100,1000,1001,1100,1101.

*For each subset S=(c,d), let $c=c_{1}\ldots c_{l}$ and $d_{1}\ldots d_{l}$. To generate the header Hdr and encryption key K, the encrypt algorithm selects a random $s\in Z_{p}$ and set Hdr and K as*

(2) $$Hdr\leftarrow (g^{s},{\left(h_{1,c_{1}}\ldots h_{l,c_{l}}k_{1,d_{1}}\ldots k_{l,d_{l}}g_{3}\right)}^{s})$$

(3) $$K\leftarrow e{\left(g_{1},g_{2}\right)}^{s}$$

where $Hdr\in G^{2}$ and $K\in G_{1}$, $h_{i,*}=h_{i,0}h_{i,1}$, and $k_{i,*}=1$.

**Example** **2.** For subset (**0*,0*01),

$$\begin{array}{c} Hdr\leftarrow (g^{s},{\left(h_{1,0}h_{1,1}h_{2,0}h_{2,1}h_{3,0}h_{4,0}h_{4,1}k_{1,0}k_{3,0}k_{4,1}g_{3}\right)}^{s}) \end{array}$$

$$K\leftarrow {\left(g_{1},g_{2}\right)}^{s}.$$

Decrypt($S,ID,SK_{ID},Hdr$): Consider an identity $ID=b_{1}\cdots b_{l}$ and a subset S=(c,d). Let j be an index where the bit in b and d is different, that is, such that $b_{j}=1$ and $d_{j}=0$, or $b_{j}=0$ and $d_{j}=1$. For example, if d=0*01 and ID=0000, then j=4 since $b_{4}=0$ and $d_{4}=1$.

From $SK_{ID}=\left(SK_{ID,1},\ldots ,SK_{ID,l}\right)$, the decrypt algorithm choose $SK_{ID,j}$ such that index j satisfies the condition stated above. To retrieve decrypt key K from the header $Hdr=(A_{0},A_{1})$ and the secret key $SK_{ID,j}=(a_{0},a_{1},h_{1,{\bar{b}}_{1}}^{r_{j}},\ldots ,h_{l,{\bar{b}}_{l}}^{r_{j}},$$k_{1,0}^{r_{j}},k_{1,1}^{r_{j}},\ldots ,k_{j-1,0}^{r_{j}},k_{j-1,1}^{r_{j}},k_{j,b_{j}}^{r_{j}}$, $k_{j+1,0}^{r_{j}},k_{j+1,1}^{r_{j}},\ldots ,k_{l,0}^{r_{j}},k_{l,1}^{r_{j}})$, let $B=a_{1}\;\cdot \;\prod_{i=1,c_{i}=*}^{l}h_{i,{\bar{b}}_{i}}^{r_{j}}\;\cdot \;\prod_{i=1,i\neq j,d_{i}\neq *}^{l}k_{i,d_{i}}^{r_{j}}$ and output

(4) $$\begin{array}{c} \frac{e(A_{0},B)}{e(a_{0},A_{1})}=K \end{array}$$

*For a valid ciphertext, it satisfies that*

$$\begin{array}{cc} \frac{e(A_{0},B)}{e(a_{0},A_{1})} & =\frac{e(g^{s},g_{2}^{\alpha }{\left(h_{1,c_{1}}\cdots h_{l,c_{l}}k_{1,d_{1}}\cdots k_{l,d_{l}}g_{3}\right)}^{r_{j}})}{e(g^{r_{j}},{\left(h_{1,c_{1}}\cdots h_{l,c_{l}}k_{1,d_{1}}\cdots k_{l,d_{l}}g_{3}\right)}^{s})}\\ & =e{\left(g,g_{2}\right)}^{s\alpha }=e{\left(g_{1},g_{2}\right)}^{s}. \end{array}$$

For the example above, with ID=0000, (c,d)=(**0*,0*01), and $Hdr=(g^{s},(h_{1,0}h_{1,1}h_{2,0}h_{2,1}h_{3,0}h_{4,0}h_{4,1}$$k_{1,0}k_{3,0}k_{4,1}g_{3}{)}^{s})$. Since $b_{4}=0$, $d_{4}=1$, and j=4, using $SK_{0000,4}$ = $\left(g^{r_{4}},g_{2}^{\alpha }{\left(h_{1,0}h_{2,0}h_{3,0}h_{4,0}k_{4,1}g_{3}\right)}^{r_{4}},\ldots \right)$, we compute *B* as follows.

$$\begin{array}{cc} B & =a_{1}\;\cdot \;{\left(h_{1,1}h_{2,1}h_{4,1}\right)}^{r_{4}}\;\cdot \;{\left(k_{1,0}k_{3,0}\right)}^{r_{4}}\\ \; & =g_{2}^{\alpha }{\left(h_{1,0}h_{2,0}h_{3,0}h_{4,0}k_{4,1}g_{3}\right)}^{r_{4}}\;\cdot \;{\left(h_{1,1}h_{2,1}h_{4,1}\right)}^{r_{4}}\;\cdot \;{\left(k_{1,0}k_{3,0}\right)}^{r_{4}}=g_{2}^{\alpha }{\left(h_{1,0}h_{1,1}h_{2,0}h_{2,1}h_{3,0}h_{4,0}h_{4,1}k_{1,0}k_{3,0}k_{4,1}g_{3}\right)}^{r_{4}}. \end{array}$$

$$\begin{array}{cc} \frac{e(A_{0},B)}{e(a_{0},A_{1})} & =\frac{e(g^{s},g_{2}^{\alpha }{\left(h_{1,0}h_{1,1}h_{2,0}h_{2,1}h_{3,0}h_{4,0}h_{4,1}k_{1,0}k_{3,0}k_{4,1}g_{3}\right)}^{r_{4}})}{e(g^{r_{4}},{\left(h_{1,0}h_{1,1}h_{2,0}h_{2,1}h_{3,0}h_{4,0}h_{4,1}k_{1,0}k_{3,0}k_{4,1}g_{3}\right)}^{s})}\\ \; & =\frac{e{\left(g,g_{2}\right)}^{s\alpha }e{\left(g,h_{1,0}h_{1,1}h_{2,0}h_{2,1}h_{3,0}h_{4,0}h_{4,1}k_{1,0}k_{3,0}k_{4,1}g_{3}\right)}^{sr_{4}}}{e{\left(g,h_{1,0}h_{1,1}h_{2,0}h_{2,1}h_{3,0}h_{4,0}h_{4,1}k_{1,0}k_{3,0}k_{4,1}g_{3}\right)}^{sr_{4}}}=e{\left(g,g_{2}\right)}^{s\alpha }. \end{array}$$

Now, let us analyze the opposite case of ID=0010, which is not included by an inclusion label **0* from the subset (**0*,0*01). Since $SK_{0010,i}=\left(g^{r_{i}},g_{2}^{\alpha }{\left(h_{1,0}h_{2,0}h_{3,1}h_{4,0}k_{i,{\bar{b}}_{i}}\right)}^{r_{i}},\ldots \right)$ and $A_{1}={\left(h_{1,0}h_{1,1}h_{2,0}h_{2,1}h_{3,0}h_{4,0}h_{4,1}\ldots \right)}^{s}$, it is necessary to eliminate $h_{3,1}^{r_{i}}$ from $SK_{0010,i}$, which is not available. Thus, it is impossible to calculate *B* using $SK_{0010,i}$.

Let us examine ID=0001 for instance, which is included in the exclusion label 0*01 of subset (**0*,0*01). Since $SK_{0001,1}=\left(g^{r_{1}},g_{2}^{\alpha }{\left(\ldots k_{1,1}\right)}^{r_{1}},\ldots \right)$, $SK_{0001,2}=\left(g^{r_{2}},g_{2}^{\alpha }{\left(\ldots k_{2,1}\right)}^{r_{2}},\ldots \right)$, $SK_{0001,3}=\left(g^{r_{3}},g_{2}^{\alpha }{\left(\ldots k_{3,1}\right)}^{r_{3}},\ldots \right)$, $SK_{0001,4}=\left(g^{r_{4}},g_{2}^{\alpha }{\left(\ldots k_{4,0}\right)}^{r_{4}},\ldots \right)$, and $A_{1}={\left(\ldots k_{1,0}k_{3,0}k_{4,1}\right)}^{s}$, it is necessary to eliminate $k_{1,1}^{r_{1}}$ from $SK_{0001,1}$, $k_{2,1}^{r_{2}}$ from $SK_{0001,2}$, $k_{3,1}^{r_{3}}$ from $SK_{0001,3}$, or $k_{4,0}^{r_{4}}$ from $SK_{0001,4}$, which is not possible. Thus, *B* cannot be computed from $SK_{0001,i}$.

Notice that the encrypt algorithm is applied for each subset *S*. In other words, the number of Hdr is equal to the number of subsets. When the user receives a message, he chooses Hdr for the set where he belongs and call Decrypt for that specific Hdr.

## 6. CPA-Security Analysis

In this section, we formally prove the semantic security (IND-CPA-security) of our CSD-based broadcast encryption in Section 5 under the decisional *l*-BDHE assumption without the random oracle model.

**Theorem** **3.** *Let G be a bilinear group of prime order p. Suppose the (decision) (t,ϵ,4l)-BDHE assumption holds in G. Then our l-level CSD public key broadcast encryption system is $\left(t^{\prime },\epsilon ,l,m\right)$ semantically secure for arbitrary l, and $t^{\prime }<t-O\left(el^{2}2^{l}\right)$, where e is the maximum time for an exponentiation in G.*

**Proof.**  Suppose that the adversary A has advantage ϵ in attacking the *l*-level CSD-based public key broadcast encryption. Using A, we build an algorithm B which solves the (decisional) 4l-BDHE problem in G.

For generators h,g∈G and $\alpha \in Z_{p}^{*}$, let $y_{i}=g^{\alpha^{i}}\in G$. As a beginning of the game, B starts a game with the 4l-BDHE problem, to get a random tuple ($h,g,y_{1},\ldots ,y_{4l},y_{4l+2},\ldots ,y_{8l},T$) which is either sampled from $P_{BDHE}$ (where $T=e{\left(h,g\right)}^{\left(\alpha^{4l+1}\right)}$) or from $R_{BDHE}$ (where *T* is uniform and independent in $G_{1}$ ). The goal of B’s is to output 1 if the tuple is sampled from $P_{BDHE}$, or output 0 otherwise. B proceeds by interacting with A in a following selective subset game:

Init: The game begins with A first outputting a subset $S^{*}=\left(c^{*},d^{*}\right)$ that it intends to attack where $c^{*}$, $d^{*}\in {\left\{0,1,*\right\}}^{l}$.

Setup: To generate the public key, algorithm B picks a random γ in $Z_{p}$ and sets $g_{1}=y_{1}={g^{\prime }}^{\alpha }$ and $g_{2}=y_{4l}g^{\gamma }=g^{\gamma +(\alpha^{4l})}$. B picks random $\gamma_{1,0},\gamma_{1,1},\cdots ,\gamma_{l,0},\gamma_{l,1}$ and $\psi_{1,0},\psi_{1,1},\cdots ,\psi_{l,0},\psi_{l,1}$ in $Z_{p}$, to set $h_{i,0}=g^{\gamma_{i,0}}/y_{l-i+1},h_{i,1}$ = $g^{\gamma_{i,1}}/y_{2l-i+1},k_{i,0}=g^{\psi_{i,0}}/y_{3l-i+1},k_{i,1}=g^{\psi_{i,1}}/y_{4l-i+1}$ for i=1,…,l. B also picks a random δ in $Z_{p}$ and sets $g_{3}=g^{\delta }\prod_{i=1}^{l}y_{l-i+1}^{c_{i,0}^{*}}y_{2l-i+1}^{c_{i,1}^{*}}y_{3l-i+1}^{d_{i,0}^{*}}y_{4l-i+1}^{d_{i,1}^{*}}$ such that $c_{i,0}^{*}=0$ and $c_{i,1}^{*}=1$ if $c_{i}^{*}=1$, $c_{i,0}^{*}=1$ and $c_{i,1}^{*}=0$ if $c_{i}^{*}=0$, and $c_{i,0}^{*}=1$ and $c_{i,1}^{*}=1$ if $c_{i}^{*}=*$. $d_{i,0}^{*}$ and $d_{i,1}^{*}$ are similarly defined by $d_{i}^{*}$ except that $d_{i,0}^{*}=0$ and $d_{i,1}^{*}=0$ if $d_{i}^{*}=*$.

Consider a query for the secret key corresponding to $ID=b_{1}\ldots b_{l}\in {\left\{0,1\right\}}^{l}$. We now show that the revoked users can be simulated with two cases as follows. Case 1 shows how to simulate users that are not in the inclusion label $c^{*}$ in subset ($c^{*},d^{*}$). Case 2 shows that we can also simulate users included in the exclusion label $d^{*}$.

**Case 1:** If *ID* 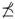 c* there exists k∈{1,…,l} such that $b_{k}\neq c_{k}^{*}$, $b_{k}\neq *$, and $c_{k}^{*}\neq *$. We set *k* to be the smallest of such index. To generate the secret key $SK_{ID,j}$, *B* first picks random ${\tilde{r}}_{1},\ldots ,{\tilde{r}}_{l}$ in $Z_{p}$. We pose $r_{j}=\alpha^{(3-b_{k})l+k}+{\tilde{r}}_{j}$ for j=1,…,l. Next, B generates the secret key $SK_{ID}=\left(SK_{ID,1},\ldots ,SK_{ID,l}\right)$ where

$$\begin{array}{c} SK_{ID,j}=\left(g^{r_{j}},g_{2}^{\alpha }{\left(h_{1,b_{1}}\ldots h_{l,b_{l}}k_{j,{\bar{b}}_{j}}g_{3}\right)}^{r_{j}},h_{1,{\bar{b}}_{1}}^{r_{j}},\ldots ,h_{l,{\bar{b}}_{l}}^{r_{j}},k_{1,0}^{r_{j}},k_{1,1}^{r_{j}},\ldots ,k_{j-1,0}^{r_{j}},k_{j-1,1}^{r_{j}},k_{j,b_{j}}^{r_{j}},k_{j+1,0}^{r_{j}},k_{j+1,1}^{r_{j}},\ldots ,k_{l,0}^{r_{j}},k_{l,1}^{r_{j}}\right) \end{array}$$

which is a properly distributed secret key for the identity $ID=b_{1}\ldots b_{l}$. B can compute all elements for the secret key, given the values at its disposal, considering the fact that $y_{i}^{\alpha^{j}}=y_{i+j}$ for any i,j. Note that $b_{i,0}={\bar{b}}_{i}$ and $b_{i,1}=b_{i}$ if $b_{i}\neq *$; otherwise, $b_{i,0}=b_{i,1}=1$. If $b_{j}=*$ and an equation includes ${\bar{b}}_{j}$ then two equations are created by replacing ${\bar{b}}_{j}$ by 0 and 1. To generate the second component of the secret key, first observe that

$$\begin{array}{c} {\left(h_{1,b_{1}}\cdots h_{l,b_{l}}k_{j,{\bar{b}}_{j}}g_{3}\right)}^{r_{j}}={\left(\prod_{i=1}^{l}h_{i,0}^{b_{i,0}}h_{i,1}^{b_{i,1}}\;\cdot \;k_{j,{\bar{b}}_{j}}\;\cdot \;g_{3}\right)}^{r_{j}}\\ ={\left(\prod_{i=1}^{l}{\left(\frac{g^{\gamma_{i,0}}}{y_{l-i+1}}\right)}^{b_{i,0}}{\left(\frac{g^{\gamma_{i,1}}}{y_{2l-i+1}}\right)}^{b_{i,1}}\;\cdot \;\frac{g^{\psi_{j,{\bar{b}}_{j}}}}{y_{(3+{\bar{b}}_{j})l-j+1}}\;\cdot \;g^{\delta }\prod_{i=1}^{l}y_{l-i+1}^{c_{i,0}^{*}}y_{2l-i+1}^{c_{i,1}^{*}}y_{3l-i+1}^{d_{i,0}^{*}}y_{4l-i+1}^{d_{i,1}^{*}}\right)}^{r_{j}}\\ ={\left(g^{\delta +\psi_{j,{\bar{b}}_{j}}+\sum_{i=1,i\neq k}^{l}\left(b_{i,0}\gamma_{i,0}+b_{i,1}\gamma_{i,1}\right)}\prod_{i=1,i\neq k}^{l}\left(y_{l-i+1}^{c_{i,0}^{*}-b_{i,0}}y_{2l-i+1}^{c_{i,1}^{*}-b_{i,1}}\right)\;\cdot \;\prod_{i=1}^{l}\left(y_{3l-i+1}^{d_{i,0}^{*}}y_{4l-i+1}^{d_{i,1}^{*}}\right)y_{3l-j+1}^{-b_{j,0}}y_{4l-j+1}^{-b_{j,1}}y_{l-k+1}^{c_{k,0}^{*}-b_{k,0}}y_{2l-k+1}^{c_{k,1}^{*}-b_{k,1}}\right)}^{r_{j}}\\ ={\left(g^{\delta +\psi_{j,{\bar{b}}_{j}}+\sum_{i=1,i\neq k}^{l}\left(b_{i,0}\gamma_{i,0}+b_{i,1}\gamma_{i,1}\right)}\prod_{i=1,i\neq k}^{l}\left(y_{l-i+1}^{c_{i,0}^{*}-b_{i,0}}y_{2l-i+1}^{c_{i,1}^{*}-b_{i,1}}\right)\;\cdot \;\prod_{i=1}^{l}\left(y_{3l-i+1}^{d_{i,0}^{*}}y_{4l-i+1}^{d_{i,1}^{*}}\right)y_{3l-j+1}^{-b_{j,0}}y_{4l-j+1}^{-b_{j,1}}y_{(1+{\bar{b}}_{k})l-k+1}^{c_{k,{\bar{b}}_{k}}^{*}}y_{(1+b_{k})l-k+1}^{-1}\right)}^{r_{j}}. \end{array}$$

Let $Z^{r_{j}}$ denote the product of *y* except the last one.

$$\begin{array}{cc} Z^{r_{j}}= & {\left(g^{\delta +\psi_{j,{\bar{b}}_{j}}+\sum_{i=1,i\neq k}^{l}\left(b_{i,0}\gamma_{i,0}+b_{i,1}\gamma_{i,1}\right)}\prod_{i=1,i\neq k}^{l}\left(y_{l-i+1}^{c_{i,0}^{*}-b_{i,0}}y_{2l-i+1}^{c_{i,1}^{*}-b_{i,1}}\right)\;\cdot \;\prod_{i=1}^{l}\left(y_{3l-i+1}^{d_{i,0}^{*}}y_{4l-i+1}^{d_{i,1}^{*}}\right)y_{3l-j+1}^{-b_{j,0}}y_{4l-j+1}^{-b_{j,1}}y_{(1+{\bar{b}}_{k})l-k+1}^{c_{k,{\bar{b}}_{k}}^{*}}\right)}^{r_{j}}\\ = & y_{(3-b_{k})l+k}^{\delta +\psi_{j,{\bar{b}}_{j}}+\sum_{i=1,i\neq k}^{l}\left(b_{i,0}\gamma_{i,0}+b_{i,1}\gamma_{i,1}\right)}\;\cdot \;\prod_{i=1,i\neq k}^{l}\left(y_{(4-b_{k})l-i+k+1}^{c_{i,0}^{*}-b_{i,0}}y_{(5-b_{k})l-i+k+1}^{c_{i,1}^{*}-b_{i,1}}\right)\;\cdot \;\prod_{i=1}^{l}\left(y_{(6-b_{k})l-i+k+1}^{d_{i,0}^{*}}y_{(7-b_{k})l-i+k+1}^{d_{i,1}^{*}}\right)\\ \; & \;\cdot \;y_{(6-b_{k})l-j+1}^{-b_{j,0}}y_{(7-b_{k})l-j+1}^{-b_{j,1}}y_{(4-b_{k}+{\bar{b}}_{k})l+1}^{c_{k,{\bar{b}}_{k}}^{*}}Z^{{\bar{r}}_{j}}. \end{array}$$

Since $(3-b_{k})l+k<4l$, $\left(4-b_{k}\right)l-i+k+1=\left(4-b_{k}\right)l+1+\left(k-i\right)\neq 4l+1$ and $(5-b_{k})l-i+k+1\neq 4l+1$ due to k≠i, $\left(7-b_{k}\right)l-i+k+1>\left(6-b_{k}\right)l-i+k+1\geq 4l+k+1>4l+1$, $\left(7-b_{k}\right)l-j+k+1>\left(6-b_{k}\right)l-j+k+1\geq 5l-l+k+1>4l+1$, $(4-b_{k}+{\bar{b}}_{k})l+1$ = 3l+1 or 5l+1, and *Z* is computable, B is able to compute all the terms in $Z^{r_{j}}$. Next, when observing the last term, $y_{(1+b_{k})l-k+1}^{-r_{j}}$ is:

$$\begin{array}{c} y_{(1+b_{k})l-k+1}^{-r_{j}}=y_{(1+b_{k})l-k+1}^{-{\tilde{r}}_{j}}y_{(1+b_{k})l-k+1}^{-\alpha^{(3-b_{k})l+k}}=y_{(1+b_{k})l-k+1}^{-{\tilde{r}}_{j}}y_{4l+1}^{-1}. \end{array}$$

Hence, the first component in the secret key is equal to:

$$\begin{array}{c} g_{2}^{\alpha }{\left(h_{1,b_{1}}\cdots h_{l,b_{l}}k_{j,{\bar{b}}_{j}}g_{3}\right)}^{r_{j}}=\left(y_{4l+1}y_{1}^{\gamma }\right)Z^{r_{j}}\left(y_{(1+b_{k})l-k+1}^{-{\tilde{r}}_{j}}y_{4l+1}^{-1}\right)=y_{1}^{\gamma }y_{(1+b_{k})l-k+1}^{-{\tilde{r}}_{j}}Z^{r_{j}}. \end{array}$$

Notice that the unknown term $y_{4l+1}$ cancels out, which indicates that B can compute the first secret key component. The first component, $g^{r_{j}}$, is $y_{(3-b_{k})l+k}g^{{\tilde{r}}_{j}}$ which can be computed by B. In a similar manner, the remaining elements $h_{1,{\bar{b}}_{1}}^{r_{j}},\ldots ,h_{l,{\bar{b}}_{l}}^{r_{j}},k_{1,0}^{r_{j}},k_{1,1}^{r_{j}},\ldots ,k_{j-1,0}^{r_{j}},k_{j-1,1}^{r_{j}}k_{j,b_{j}}^{r_{j}},k_{j+1,0}^{r_{j}}$, $k_{j+1,1}^{r_{j}},\ldots ,k_{l,0}^{r_{j}},k_{l,1}^{r_{j}}$ can be computed by B since they do not involve a $y_{4l+1}$ term such that $h_{i,{\bar{b}}_{i}}^{r_{j}}={\left(\frac{g^{\gamma_{i,{\bar{b}}_{i}}}}{y_{(1+{\bar{b}}_{i})l-i+1}}\right)}^{r_{j}}$$={\left(y_{(3-b_{k})l+k}\;\cdot \;g^{{\tilde{r}}_{j}}\right)}^{\gamma_{i,{\bar{b}}_{i}}}\;\cdot \;{\left(y_{(4-b_{k}+{\bar{b}}_{i})l-i+k+1}y_{(1+{\bar{b}}_{i})l-i+1}^{{\tilde{r}}_{j}}\right)}^{-1}$ and $k_{i,t}^{r_{j}}={\left(\frac{g^{\psi_{i,t}}}{y_{(3+t)l-i+1}}\right)}^{r_{j}}={\left(y_{(3-b_{k})l+k}\;\cdot \;g^{{\tilde{r}}_{j}}\right)}^{\psi_{i,t}}\;\cdot \;{\left(y_{(6-b_{k}+t)l-i+k+1}\;\cdot \;y_{(3+t)l-i+1}^{{\tilde{r}}_{j}}\right)}^{-1}$. Thus, B can derive $h_{i,{\bar{b}}_{i}}^{r_{j}}$ since $\left(4-b_{k}+{\bar{b}}_{i}\right)l-i+k+1\neq \left(4-b_{k}+{\bar{b}}_{i}\right)l+1$ when i≠k and $(4-b_{k}+{\bar{b}}_{i})l-i+k+1$ = 3l+1 or 5l+1 when i=k. Moreover, B can derive $k_{i,t}^{r_{j}}$ since $(3-b_{k})l+k\leq 4l$, $(6-b_{k}+t)l-i+k+1\geq 5l-i+k+1>4l+1$, and (3+t)l−i+1≤4l.

**Case 2:** If $ID⪯d^{*}$, to generate the secret key $SK_{ID,j}$ for identity $ID=b_{1}\ldots b_{l}$, B first selects a random ${\tilde{r}}_{1},\ldots ,{\tilde{r}}_{l}$ in $Z_{p}$. We pose $r_{j}=\alpha^{(1-{\bar{b}}_{j})l+j}+{\tilde{r}}_{j}$ for j=1,…,l. Next, B generates the secret key $SK_{ID}=\left(SK_{ID,1},\ldots ,SK_{ID,l}\right)$ The secret key parameters are computed similarly. To generate the second component of the secret key, first observe that

$$\begin{array}{cc} \; & {\left(h_{1,b_{1}}\cdots h_{l,b_{l}}k_{j,{\bar{b}}_{j}}g_{3}\right)}^{r_{j}}\\ \; & \;\;\;\;\;={\left(g^{\delta +\psi_{j,{\bar{b}}_{j}}+\sum_{i=1}^{l}\left(b_{i,0}\gamma_{i,0}+b_{i,1}\gamma_{i,1}\right)}\prod_{i=1}^{l}\left(y_{l-i+1}^{c_{i,0}^{*}-b_{i,0}}y_{2l-i+1}^{c_{i,1}^{*}-b_{i,1}}\right)\prod_{i=1,i\neq j}^{l}\left(y_{3l-i+1}^{d_{i,0}^{*}}y_{4l-i+1}^{d_{i,1}^{*}}\right)\;\cdot \;y_{(3+b_{j})l-j+1}^{d_{j,b_{j}}^{*}}\;\cdot \;y_{(3+{\bar{b}}_{j})l-j+1}^{-1}\right)}^{r_{j}}. \end{array}$$

Let $Z^{r_{j}}$ denote the product of *y* except the last one. That is

$$\begin{array}{c} Z^{r_{j}}=(g^{\delta +\psi_{j,{\bar{b}}_{j}}+\sum_{i=1}^{l}\left(b_{i,0}\gamma_{i,0}+b_{i,1}\gamma_{i,1}\right)}\prod_{i=1}^{l}\left(y_{l-i+1}^{c_{i,0}^{*}-b_{i,0}}y_{2l-i+1}^{c_{i,1}^{*}-b_{i,1}}\right)\prod_{i=1,i\neq j}^{l}\left(y_{3l-i+1}^{d_{i,0}^{*}}y_{4l-i+1}^{d_{i,1}^{*}}\right)\;\cdot \;y_{(3+b_{j})l-j+1}^{d_{j,b_{j}}^{*}}{)}^{r_{j}}. \end{array}$$

B can compute all the terms in $Z^{r_{j}}$ given the values at its disposal since $l-i+1+\left(1-{\bar{b}}_{j}\right)l+j\leq \left(2-{\bar{b}}_{j}\right)l-i+j+1\leq 3l$, $2l-i+1+(1-{\bar{b}}_{j})l+j\leq 4l$, $3l-i+1+\left(1-{\bar{b}}_{j}\right)l+j=\left(4-{\bar{b}}_{j}\right)l-i+j+1\neq 4l+1$, $4l-i+1+\left(1-{\bar{b}}_{j}\right)l+j=\left(5-{\bar{b}}_{j}\right)l-i+j+1\neq 4l+1$ due to i≠j, and $\left(3+b_{j}\right)l-j+1+\left(1-{\bar{b}}_{j}\right)l+j$ = $(4+b_{j}-{\bar{b}}_{j})l+1$ = 3l+1 or 5l+1. Note that if $b_{j}=*$ then $d_{j,0}^{*}=d_{j,1}^{*}=0$, and each case that $b_{j}=0$ or $b_{j}=1$ is considered. Next observe that the last term, namely $y_{(3+{\bar{b}}_{j})l-j+1}^{-r_{j}}$, is:

$$\begin{array}{c} y_{(3+{\bar{b}}_{j})l-j+1}^{-r_{j}}=y_{(3+{\bar{b}}_{j})l-j+1}^{-{\tilde{r}}_{j}}y_{(3+{\bar{b}}_{j})l-j+1}^{-\alpha^{(1-{\bar{b}}_{j})l+j}}=y_{(3+{\bar{b}}_{j})l-j+1}^{-{\tilde{r}}_{j}}\;\cdot \;y_{4l+1}^{-1}. \end{array}$$

Therefore, the first component in the secret key is equal to:

$$\begin{array}{c} g_{2}^{\alpha }{\left(h_{1,b_{1}}\ldots h_{l,b_{l}}k_{j,{\bar{b}}_{j}}g_{3}\right)}^{r_{j}}=\left(y_{4l+1}y_{1}^{\gamma }\right)Z^{r_{j}}\left(y_{(3+{\bar{b}}_{j})l-j+1}^{-{\tilde{r}}_{j}}/y_{4l+1}\right)=y_{1}^{\gamma }y_{(3+{\bar{b}}_{j})l-j+1}^{-{\tilde{r}}_{j}}Z^{r_{j}}. \end{array}$$

Notice that the unknown $y_{4l+1}$ cancels out, which indicates that B can compute the first secret key component. The first component, $g^{r_{j}}$, is $y_{(1-{\bar{b}}_{j})l+j}g^{{\tilde{r}}_{j}}$ which can be computed by B. In a similar manner, B can compute the remaining elements $h_{1,{\bar{b}}_{1}}^{r_{j}},\ldots$,

$h_{l,{\bar{b}}_{l}}^{r_{j}},k_{1,0}^{r_{j}},k_{1,1}^{r_{j}},\ldots$, $k_{j-1,0}^{r_{j}},k_{j-1,1}^{r_{j}},k_{j,b_{j}}^{r_{j}},k_{j+1,0}^{r_{j}},k_{j+1,1}^{r_{j}},\ldots$, $k_{l,0}^{r_{j}},k_{l,1}^{r_{j}}$ since they do not involve $y_{4l+1}$ as $h_{i,{\bar{b}}_{i}}^{r_{j}}={\left(\frac{g^{\gamma_{i,{\bar{b}}_{i}}}}{y_{(1+{\bar{b}}_{i})l-i+1}}\right)}^{r_{j}}$ $={\left(y_{(1-{\bar{b}}_{j})l+j}\;\cdot \;g^{{\tilde{r}}_{j}}\right)}^{\gamma_{i,{\bar{b}}_{i}}}\;\cdot \;{\left(y_{(2+{\bar{b}}_{i}-{\bar{b}}_{j})l-i+j+1}y_{(1+{\bar{b}}_{i})l-i+1}^{{\tilde{r}}_{j}}\right)}^{-1}$ and $k_{i,t}^{r_{j}}={\left(\frac{g^{\psi_{i,t}}}{y_{(3+t)l-i+1}}\right)}^{r_{j}}$ = ${\left(y_{(1-{\bar{b}}_{j})l+j}\;\cdot \;g^{{\tilde{r}}_{j}}\right)}^{\psi_{i,t}}\;\cdot \;{\left(y_{(4+t-{\bar{b}}_{j})l-i+j+1}\;\cdot \;y_{(3+t)l-i+1}^{{\tilde{r}}_{j}}\right)}^{-1}$. Thus, B can derive a valid secret key for ID since $\left(4+t-{\bar{b}}_{j}\right)l-i+j+1\neq \left(4+t-{\bar{b}}_{j}\right)l+1$ when i≠j and $(4-t+{\bar{b}}_{j})l-i+j+1=3l+1$ or 5l+1 due to $t=b_{j}$ when i=j.

Therefore, B can derive a valid secret key for ID. Finally, B gives A public key PK = ($g,g_{1},g_{2},g_{3},h_{1,0},h_{1,1},$$\cdots ,h_{l,0},h_{l,1}$, $k_{1,0},k_{1,1},\cdots ,k_{l,0},k_{l,1}$) and $SK_{ID}$ such that *ID* 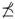 c* or $ID⪯d^{*}$. All the values are within an independent and uniform distribution from G, as required. The master key for the corresponding system is supposed to be $g_{2}^{\alpha }=g^{\alpha (\alpha^{4l}+\gamma )}=y_{4l+1}y_{1}^{\gamma }$, which is not known to B since B does not know $y_{4l+1}$.

For the challenge, B generates $Hdr^{*}$ as $\left(h,h^{\delta +\sum_{i=1}^{l}\left(\gamma_{i,0}c_{i,0}^{*}+\gamma_{i,1}c_{i,1}^{*}+\psi_{i,0}d_{i,0}^{*}+\psi_{i,1}d_{i,1}^{*}\right)}\right)$. It sets $K^{*}=T\;\cdot \;e\left(y_{1},h^{\gamma }\right)$ and gives $\left(Hdr^{*},K^{*}\right)$ to A, as a challenge. If $T=e{\left(g,h\right)}^{4l+1}$ (i.e., the input to B is from the 4l-BDHE) then the challenge $\left(Hdr^{*},K^{*}\right)$ is valid from *A*’s view as in the real attacking game. This can be seen by writing $h=g^{c}$ for some (unknown) $c\in Z_{p}$ as follows:

$$\begin{array}{cc} \; & h^{\delta +\sum_{i=1}^{l}\left(\gamma_{i,0}c_{i,0}^{*}+\gamma_{i,1}c_{i,1}^{*}+\psi_{i,0}d_{i,0}^{*}+\psi_{i,1}d_{i,1}^{*}\right)}\\ \; & \;\;\;\;\;={\left(\prod_{i=1}^{l}{\left(\frac{g^{\gamma_{i,0}}}{y_{l-i+1}}\right)}^{c_{i,0}^{*}}{\left(\frac{g^{\gamma_{i,1}}}{y_{2l-i+1}}\right)}^{c_{i,1}^{*}}{\left(\frac{g^{\psi_{i,0}}}{y_{3l-i+1}}\right)}^{d_{i,0}^{*}}{\left(\frac{g^{\psi_{i,1}}}{y_{4l-i+1}}\right)}^{d_{i,1}^{*}}\;\cdot \;\left(g^{\delta }\prod_{i=1}^{l}y_{l-i+1}^{c_{i,0}^{*}}y_{2l-i+1}^{c_{i,1}^{*}}y_{3l-i+1}^{d_{i,0}^{*}}y_{4l-i+1}^{d_{i,1}^{*}}\right)\right)}^{c}\\ \; & \;\;\;\;\;={\left(h_{1,c_{1}^{*}}\cdots h_{l,c_{l}^{*}}k_{1,d_{1}^{*}}\cdots k_{l,d_{l}^{*}}g_{3}\right)}^{c} \end{array}$$

and

$$\begin{array}{cc} \; & e{\left(g,h\right)}^{\left(\alpha^{4l+1}\right)}\;\cdot \;e\left(y_{1},h^{\gamma }\right)={\left(e\left(y_{1},y_{4l}\right)\;\cdot \;e\left(y_{1},g^{\gamma }\right)\right)}^{c}=e{\left(y_{1},y_{4l}g^{\gamma }\right)}^{c}=e{\left(g_{1},g_{2}\right)}^{c}. \end{array}$$

Thus, by definition, $Hdr^{*}$ is a valid encryption of $e{\left(y_{4l+1},g\right)}^{c}$. Also, observe that $e{\left(y_{4l+1},g\right)}^{c}=e{\left(g,h\right)}^{4l+1}=T=K_{b}$, which confirms that ($Hdr,K^{*}$) is a valid challenge to the A. On the other hand, if *T* is random in G (i.e., the input to B is a random tuple), then $K^{*}$ is just a random independent key of K from A’s view.

Guess:

Finally, A outputs a guess $b^{\prime }\in \left\{0,1\right\}$. Algorithm B concludes its own game by outputting the $b^{\prime }$. If $b^{\prime }=1$ then B outputs 1 meaning $T=e{\left(g,h\right)}^{\left(\alpha^{4l+1}\right)}$. Otherwise, it outputs 0 meaning *T* is random in $G_{T}$.

If the input tuple was originated from $P_{BDHE}$ ($T=e{\left(g,h\right)}^{\left(\alpha^{4l+1}\right)}$), A’s view is the same as the view in a real attacking game. Therefore, A satisfies $|Pr[b^{\prime }=1]-1/2|\geq \epsilon$. When the input tuple was originated from $R_{BDHE}$ (*T* is uniform in $G_{1}$) then $Pr[b^{\prime }=1]=1/2$. Hence, with g,h uniform in G, α uniform in $Z_{p}$, and *T* uniform in $G_{1}$ we have that

(5) $$\begin{array}{c} |Pr\left[B\left(g,h,{\vec{y}}_{g,\alpha ,4l},e{\left(g,h\right)}^{\left(\alpha^{4l+1}\right)}\right)=0\right]-Pr\left[B\left(g,h,{\vec{y}}_{g,\alpha ,4l},T\right)=0\right]\left|\geq \right|\left(1/2+\epsilon \right)-1/2|=\epsilon . \end{array}$$

as required, which completes the proof of the theorem. □

## 7. CCA-secure Broadcast Encryption

In this section, we extend our proposed CPA-secure combinatorial subset difference public key broadcast encryption scheme to obtain CCA-security by using similar technique from Reference [33] which transforms a CPA-secure identity-based encryption to the CCA-secure encryption. However, to apply the similar technique, it is required to allow wildcards (*) in the ID (as well as the subset) since the technique is based on the identity-based encryption. Therefore, we first construct a general ID construction, which is a generalized version of our CSD-based broadcast encryption which allows wildcards in the user ID in Section 7.1. Then, based on the general ID construction, we build a CCA-secure CSD-based public key broadcast encryption in Section 7.2. Finally, we provide a formal proof for the CCA-security in Section 8.

### 7.1. General ID Construction

In this section, we extend the proposed broadcast encryption scheme to a generalized ID version, which allows * in each ID (as well as subsets). This general ID scheme is a basic building block for building a CCA-secure broadcast encryption in Section 7.

For labels *x* and *y*, we define x⪯y to indicate that *x* is covered by *y*, and *x* 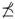 *y* to denote that *x* is not covered by *y*. For instance, 0*00⪯0**0 and 0*00 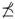 1**0.

**Definition** **5.** x is covered by y, or x⪯y iff ∀i, $x_{i}=y_{i}$ or $y_{i}=*$ for $x=x_{1}\ldots x_{l}$ and $y=y_{1}\ldots y_{l}$.

**Definition** **6.** *x is not covered by y, or *x*
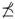
*y* iff ∀a⪯x, ∃i, $a_{i}\neq y_{i}$, $x_{i}\neq *$, and $y_{i}\neq *$.*

Setup(*l*,*m*): The setup is similar to the main scheme. The generation of public key is equivalent and the generation of secret key is similar to Equation (1), except that $h_{i,*}=1$ and $h_{i,\bar{*}}^{r_{j}}$ populates two values of $h_{i,0}^{r_{j}}$ and $h_{i,1}^{r_{j}}$. Similarly, since interpretation of $k_{i,*}$ covers both $k_{i,0}$ and $k_{i,1}$, the secret key $SK_{ID,i}$ should be doubled into $SK_{ID,i,0}$ and $SK_{ID,i,1}$ if $b_{i}=*$. The resulting secret key for ID is $SK_{ID}=\left(SK_{ID,1},\ldots ,SK_{ID,l}\right)$ where if $b_{i}\neq *$ then *i*-th secret key is the same as the key generation in the main scheme:

$$\begin{array}{cc} SK_{ID,i}= & (g^{r_{i}},g_{2}^{\alpha }{\left(h_{1,b_{1}}\cdots h_{l,b_{l}}k_{i,{\bar{b}}_{i}}g_{3}\right)}^{r_{i}},\\ & h_{1,{\bar{b}}_{1}}^{r_{i}},\ldots ,h_{l,{\bar{b}}_{l}}^{r_{i}},k_{1,0}^{r_{i}},k_{1,1}^{r_{i}},\ldots ,k_{i-1,0}^{r_{i}},k_{i-1,1}^{r_{i}},k_{i,b_{i}}^{r_{i}},k_{i+1,0}^{r_{i}},k_{i+1,1}^{r_{i}},\ldots ,k_{l,0}^{r_{i}},k_{l,1}^{r_{i}})\in G^{3l+1} \end{array}$$

else if $b_{i}=*$ then *i*-th secret key duplicated into two elements as follows: $SK_{ID,i}=\left(SK_{ID,i,0},SK_{ID,i,1}\right)$ with

(6) $$\begin{array}{cc} SK_{ID,i,0}= & (g^{r_{i,0}},g_{2}^{\alpha }{\left(h_{1,b_{1}}\cdots h_{i-1,b_{i-1}}h_{i+1,b_{i+1}}\cdots h_{l,b_{l}}k_{i,0}g_{3}\right)}^{r_{i,0}},\\ \; & h_{1,{\bar{b}}_{1}}^{r_{i,0}},\ldots ,h_{i,0}^{r_{i,0}},h_{i,1}^{r_{i,0}},\ldots ,h_{l,{\bar{b}}_{l}}^{r_{i,0}},k_{1,0}^{r_{i,0}},k_{1,1}^{r_{i,0}},\ldots ,k_{i-1,0}^{r_{i,0}},k_{i-1,1}^{r_{i,0}},k_{i,1}^{r_{i,0}},k_{i+1,0}^{r_{i,0}},k_{i+1,1}^{r_{i,0}},\ldots ,k_{l,0}^{r_{i,0}},k_{l,1}^{r_{i,0}})\\ SK_{ID,i,1}= & (g^{r_{i,1}},g_{2}^{\alpha }{\left(h_{1,b_{1}}\cdots h_{i-1,b_{i-1}}h_{i+1,b_{i+1}}\cdots h_{l,b_{l}}k_{i,1}g_{3}\right)}^{r_{i,1}},h_{1,{\bar{b}}_{1}}^{r_{i,1}},\ldots ,h_{i,0}^{r_{i,1}},\\ \; & h_{i,1}^{r_{i,1}},\ldots ,h_{l,{\bar{b}}_{l}}^{r_{i,1}},k_{1,1}^{r_{i,1}},k_{1,1}^{r_{i,1}},\ldots ,k_{i-1,0}^{r_{i,1}},k_{i-1,1}^{r_{i,1}},k_{i,0}^{r_{i,1}},k_{i+1,0}^{r_{i,1}},k_{i+1,1}^{r_{i,1}},\ldots ,k_{l,0}^{r_{i,1}},k_{l,1}^{r_{i,1}}). \end{array}$$

Encrypt(PK,*S*): Equivalent to Equations (2) and (3).

Decrypt(*S*,ID,$SK_{ID}$,Hdr): Consider an identity $ID=b_{1}\ldots b_{l}\in {\left\{0,1,*\right\}}^{l}$ and a subset S=(c,d). If ID⪯c and *ID* 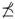 *c* then ID can decrypt the message. Let *j* be an index such that $b_{j}=*$ and $d_{j}\neq *$, $b_{j}=0$ and $d_{j}=1$, or $b_{j}=1$ and $d_{j}=0$. If $b_{j}\neq *$ then the decryption is equivalent to Equation (4). Assume that $b_{j}=*$. From $SK_{ID,j}$, we use $SK_{ID,j,d_{j}}$ as secret key. To regenerate decrypt key *K* using the given header $Hdr=(A_{0},A_{1})$ and the secret key $SK_{ID,j,d_{j}}$, compute $B=a_{1}\;\cdot \;\prod_{i=1,c_{i}=*,b_{i}\neq *}^{l}h_{i,{\bar{b}}_{i}}^{r_{j}}\prod_{i=1,c_{i}\neq *,b_{i}=*}^{l}h_{i,c_{i}}^{r_{j}}\prod_{i=1,c_{i}=*,b_{i}=*}^{l}h_{i,0}^{r_{j}}h_{i,1}^{r_{j}}\;\cdot \;\prod_{i=1,i\neq j,d_{i}\neq *}^{l}k_{i,d_{i}}^{r_{j}}$ and output

$$\begin{array}{c} \frac{e(A_{0},B)}{e(a_{0},A_{1})}=K. \end{array}$$

### 7.2. CCA-Secure Construction

For the following description, a vector $V=(v_{1},\ldots ,v_{n})$ is represented interchangeably as $v_{1}\ldots v_{n}$. With two vectors $V=(v_{1},\ldots ,v_{n})$ and $V^{\prime }=\left(v_{1}^{\prime },\ldots ,v_{m}^{\prime }\right)$, we denote $V||V^{\prime }=\left(v_{1},\ldots ,v_{n},v_{1}^{\prime },\ldots ,v_{m}^{\prime }\right)$.

Given a strong one-time signature scheme (SigKeygen,Sign,Verify) with verification keys which are mapped to ${\left\{0,1\right\}}^{z}$, we enable construction of an *l*-level public key broadcast encryption system Π=(Setup,Encrypt,Decrypt) secure against chosen-ciphertext attacks using the (l+z)-level $\Pi^{\prime }=\left({\mathrm{Setup}}^{\prime },{\mathrm{Encrypt}}^{\prime },{\mathrm{Decrypt}}^{\prime }\right)$ semantically secure broadcast encryption scheme. The intuition is that $ID=\left(b_{1},\ldots ,b_{l}\right)\in {\left\{1,0,*\right\}}^{l}$ in Π is mapped to $ID^{\prime }=ID\left|\right|{*}^{z}=\left(b_{1},\ldots ,b_{l},*,\ldots ,*\right)\in {\left\{1,0,*\right\}}^{l+z}$ in $\Pi^{\prime }$. Thus, the secret key $SK_{ID}$ for ID in Π is the secret key $SK_{ID^{\prime }}$ in $\Pi^{\prime }$. Recall that $SK_{ID||{*}^{z}}^{\prime }$ can generate secret keys of all descendants of node $ID||{*}^{z}$, that is, $SK_{ID||0^{z}}^{\prime },\ldots ,SK_{ID||1^{z}}^{\prime }$. When encrypting a key K∈K to ID in Π, the sender generates a *z*-bit verification key $V_{sig}=\left(e_{1},\ldots ,e_{z}\right)\in {\left\{0,1\right\}}^{z}$ and then encrypts *K* to the $ID^{\prime }=ID\left|\right|V_{sig}$ using $\Pi^{\prime }$.

In more details, *l*-level Π is constructed using (l+z)-level $\Pi^{\prime }$ and an one-time signature scheme as follows:

Setup(l,m): Let $2^{l}$ be the maximum number of users and ${\left\{0,1\right\}}^{m}$ be the message space. Assume the signature verification key space as ${\left\{0,1\right\}}^{z}$. To obtain the public key and master secret key, run semantically secure broadcast encryption $\Pi^{\prime }$.

$$\begin{array}{cc} \; & PK,master-key,{\left\{SK_{ID^{\prime }}^{\prime }\right\}}_{ID^{\prime }\in {\left\{0,1\right\}}^{l+z}}\leftarrow {\mathrm{Setup}}^{\prime }\left(l+z\right). \end{array}$$

To generate secret key $SK_{ID}$ for an identity $ID=b_{1}\ldots b_{l}$ using the master secret, encode ID to $ID^{\prime }=ID\left|\right|\underset{z}{\underset{︸}{**\ldots *}}$. The secret key $SK_{ID^{\prime }}^{\prime }$ is generated from the key generation algorithm in ${\mathrm{Setup}}^{\prime }$ of $\Pi^{\prime }$. Let $SK_{ID}=SK_{ID^{\prime }}^{\prime }=\left(SK_{ID^{\prime },1}^{\prime },\ldots ,SK_{ID^{\prime },l}^{\prime }\right)$. and output $PK,master-key,{\left\{SK_{ID}\right\}}_{ID\in {\left\{0,1\right\}}^{l}}$

Encrypt(PK,S): Run $SigKeyGen(1^{z})$ algorithm to receive a signature signing key $K_{sig}$ and a verification key $V_{sig}$. Assume that $V_{sig}=e_{1}\ldots e_{z}$. For a given S=(c,d), run Encrypt’ to obtain header Hdr and encryption key *K*

$$Hdr,K\leftarrow {\mathrm{Encrypt}}^{\prime }\left(PK,S\right)$$

and output the pair (Hdr,K).

Decrypt($S,ID,SK_{ID},Hdr$): Parse the header as $Hdr=(\left(C_{0},C_{1}\right),\sigma ,V_{sig})$.

1. Verify the signature σ, for $\left(C_{0},C_{1}\right)$ and $V_{sig}$. If the signature is invalid, output ⊥.
2. Otherwise, let ID to $ID^{\prime }=ID\left|\right|\underset{z}{\underset{︸}{**\ldots *}}$, run Decrypt’($S,ID^{\prime },SK_{ID},Hdr)$ and output encryption key *K*.

In a similar way, if ID contains * the extension in Section 7.1 is applied.

The correctness is straightforward from a similar calculation as in Section 5. Note that the user key size increases from $O(l^{2})$ to O((l+z)l) and the header size is enlarged by the size of a signature and a verification key.

## 8. CCA-Security Analysis

In this section, we formally prove the security against the chosen-ciphertext attack (CCA-security) of the CCA-secure CSD-based broadcst encryption in Section 7 assuming the presence of a one-time signature scheme.

**Theorem** **4.** *Let G be a bilinear group of prime order p. For all positive integer l, the above public key broadcast encryption *Π* is $\left(t,\epsilon_{1}+\epsilon_{2},l,m,q_{D}\right)$ CCA-secure when assuming the public key broadcast encryption $\Pi^{\prime }$ is $\left(t^{\prime },\epsilon_{1},l+z,m,0\right)$ semantically secure in G and the signature scheme is $\left(t^{\prime \prime },\epsilon_{2},z,1\right)$ strongly and existentially unforgeable. And $t<t^{\prime }-\left(2\left(l+z\right)a+2p\right)q_{D}-t_{s}$, where a is point addition time, p is pairing time, and $t_{s}$ is sum of SigKeyGen, Sign and Verify computation time.*

**Proof.**  Suppose there exists a *t*-time adversary, A, such that $|AdvBr_{A,\Pi }-1/2|>\epsilon_{1}+\epsilon_{2}$. We build an algorithm B, which has an advantage $|AdvBr_{B,\Pi^{\prime }}-1/2|>\epsilon_{1}$ in G. Algorithm B proceeds as follows.

Init: Algorithm B runs A and receives set $S^{*}$ in which users A wishes to be challenged on. And B runs the SigKeyGen to get a signature signing key $K_{sig}^{*}$ and a verification key $V_{sig}^{*}\in {\left\{0,1\right\}}^{z}$. Let $V_{sig}^{*}=e_{1}\ldots e_{z}$, then B makes $S^{**}$ = $\left\{U||V_{sig}^{*}\;|\;U\in S^{*}\right\}$ and outputs it.

Setup:(l,m)B gets the public key PK of $\Pi^{\prime }$ and also gets secret keys $SK_{ID^{\prime }}$ for revoked $ID^{\prime }\notin S^{**}$ from challenger C. Note that $ID^{\prime }\notin S^{**}$ iff ∀x such that $x⪯ID^{\prime }$, $x\notin S^{**}$. In addition, $ID\in S^{*}$ iff ∀x such that x⪯ID, $x\in S^{*}$.

Since $\Pi^{\prime }$ can generate secret keys in a compressed manner using *, wlog, $ID^{\prime }$ can be categorized into the following two formats:

1. $ID^{\prime }=ID\left|\right|\underset{z}{\underset{︸}{**\ldots *}}$ for $ID\notin S^{*}$
2. $ID^{\prime }=ID\left|\right|\underset{k-1}{\underset{︸}{*\ldots *}}{\bar{e}}_{k}\underset{z-k}{\underset{︸}{*\ldots *}}$ for $ID\in S^{*}$ and k∈{1,…,z}.

B responds with PK and secret keys $SK_{ID^{\prime }}^{\prime }$ of the first type of $ID^{\prime }$. (Recall that the secret key $SK_{ID}=SK_{ID^{\prime }}^{\prime }$ where $ID^{\prime }=ID\left|\right|\underset{z}{\underset{︸}{**\ldots *}}$.) The secret keys $SK_{ID^{\prime }}^{\prime }$ of the second type of $ID^{\prime }$ are used to respond to the decryption queries of A as described in the below.

Query phase1:

A can adaptively issue decryption queries. The decryption query consists of (ID,S,Hdr), where $S\subseteq S^{*}$, ID∈S, and $Hdr=(\left(C_{0},C_{1}\right),\sigma ,V_{sig})$. For the query, B replies as follows:

1. Run Verify to check the validity of signature σ on $\left(C_{0},C_{1}\right)$ by using the verification key $V_{sig}$. If the signature is not valid, B replies with ⊥.
2. If $V_{sig}=V_{sig}^{*}$, an event *forge* happens, B outputs a random bit $b\overset{\$}{\leftarrow }\left\{0,1\right\}$, and aborts the simulation.
3. Otherwise, B decrypts the header using the second type of secret keys. Let $V=\underset{k-1}{\underset{︸}{*\ldots *}}{\bar{e}}_{k}^{*}\underset{z-k}{\underset{︸}{*\ldots *}}$ where k∈{1,…,z}. Since $V_{sig}\neq V_{sig}^{*}$, $V_{sig}⪯V$. Hence, B can compute $SK_{ID||V_{sig}}$ from $SK_{ID||V}$. Using $SK_{ID||V_{sig}}$, *B* can regenerate $K\leftarrow {\mathrm{Decrypt}}^{\prime }(S$, ID, $SK_{ID||V_{sig}}$, $\left(C_{0},C_{1})\right)$.

Challenge: B gets the challenge $\left(Hdr,K^{*}\right)$ from C. To generate challenge for A, B computes $Hdr^{*}$ as follows:

$$\begin{array}{c} \sigma^{*}\leftarrow Sign\left(Hdr,K_{sig}^{*}\right)\;\;Hdr^{*}\leftarrow \left(Hdr,\sigma^{*},V_{sig}^{*}\right) \end{array}$$

B replies with $\left(Hdr^{*},K^{*}\right)$ to A.

Query phase2: Same as in query phase1.

Guess: The A outputs a guess b∈{0,1}. B outputs *b*.

We see that algorithm B can simulate all queries to run A. B’s success probability as follows:

$$\begin{array}{cc} |AdvBr_{B,\Pi^{\prime }}-\frac{1}{2}| & \geq |AdvBr_{A,\Pi }-\frac{1}{2}|-Pr\left[\mathrm{forge}\right]>\left(\epsilon_{1}+\epsilon_{2}\right)-Pr\left[\mathrm{forge}\right]. \end{array}$$

To conclude the proof, it is required to bound the probability of B aborting the simulation from *forge*. It can be seen that $Pr\left[\mathrm{forge}\right]<\epsilon_{2}$; otherwise, the adversary A can be utilized to forge signatures with a probability of at least $\epsilon_{2}$. In brief, we can construct another simulator which knows the secret key, but receives $K_{sig}^{*}$ for the challenge in a forgery game. In the experiment above, A causes an abort by submitting a query which includes an existential forgery under $K_{sig}^{*}$ on some ciphertexts. Our simulator can use A to win the existential forgery game. During the game, the adversary makes only a single chosen message query to generate the signature required for the challenge ciphertext. Thus, $Pr\left[\mathrm{forge}\right]<\epsilon_{2}$, which concludes that B’s advantage is at least $\epsilon_{1}$ as required. □

## 9. Experiments

In this section, we show the experimental results on the proposed combinatorial subset difference (CSD), and compare the result to the other public key broadcast encryption. We implemented our CSD subset representation and CSD-based broadcast encryption, and also implemented the subset difference (SD) [7,8] and interval [10] representation along with its broadcast encryption on the Intel Edison IoT device with a 500 Mhz 32 bit Atom processor. Specifically, the SD-based broadcast encryption is implemented as a public key broadcast encryption by applying the public key lifting transformation [5], which combines the hierarchical identity-based encryption (HIBE) [6] to the original symmetric Naor SD construction [7,8] from the advanced access content system (AACS) DVD standard [9]. For the comparison, we first show the number of subsets generated from each algorithm to confirm the efficiency of CSD subset representation. We also verify that the CSD-based broadcast encryption achieves the minimal header size, due to the least number of subsets. Then for the broadcast encryption, we observe the practicality of our CSD-based broadcast encryption by comparing the key size and the performance (i.e., encryption time and decryption time) to the other constructions.

**Subset Representation.** The proposed CSD subset representation is the most general representation among the existing subset representations. Therefore, it can cover more users within a subset, which decreases the number of total subsets. When considering the IoT application, the improvement is more emphasized; the non-hierarchical IP address generates numerous subsets in the existing hierarchy-based representations (e.g., SD or interval), while the CSD can cover the non-hierarchical IP address in a single representation. For example, for a binary IP address 0**.**1.***.001, existing representation methods end up generating many subsets, while the CSD can efficiently cover it with a single subset.

**Figure 6:**
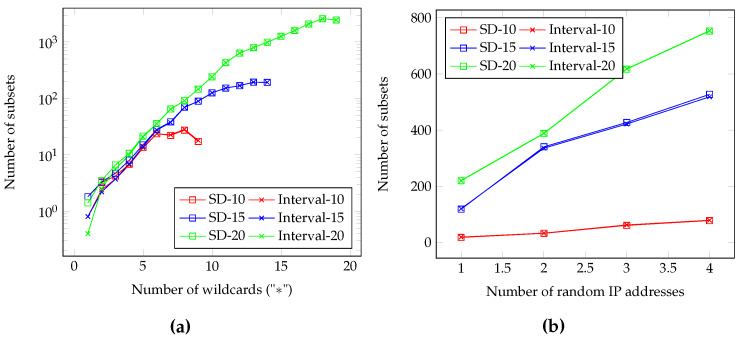
The number of subsets in IP address multicast: (**a**) for a single binary IP address (**b**) for multiple addresses.

Figure 6 shows the subset generations in the IP address application: Figure 6a presents the number of subsets when randomly increasing the number of wildcards in a single binary IP address, and Figure 6b presents the number of subsets when increasing the random IP addresses. The suffix of representations denotes a total bit of a user: for example, SD−10 indicates an SD representation for 10-bit users, or $2^{10}$ total users. In both (a) and (b), the CSD representation can cover an IP address with a single subset regardless of the total users. When observing (a), the number of subsets in SD and interval representation when the IP address includes more wildcards or the total users increase, while the CSD can cover the address within a single subset in any case. When assuming a binary IP address with 20 wildcards in $2^{20}$ total users, the CSD can remain a single subset while the SD requires more than 1000 subsets; the CSD can save the header size 1000 times compared to the current standard SD representation. In (b), the SD and interval representation generates more subsets for more IP addresses or more total users, while the number of CSD subsets are same as the number of IP addresses.

**Header Size.** Since the CSD representation generates minimal subsets, the CSD-based broadcast encryption achieves minimal (total) header sizes when applied to the broadcast encryption. We verify the result by implementing and running the broadcast encryption constructions on the IoT machine, and measuring the size of the total header output size.

**Figure 7:**
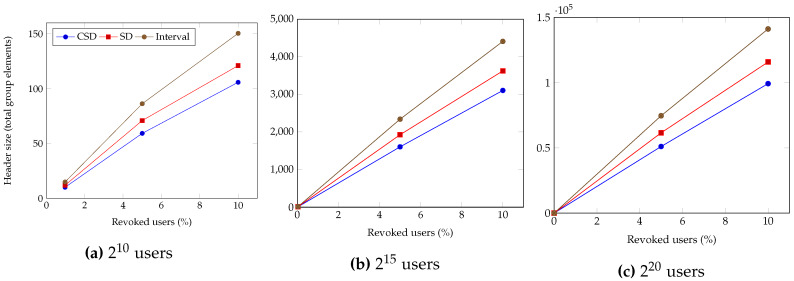
Comparison of header sizes in broadcast encryption.

Figure 7 compares the header size of each broadcast encryption, by randomly revoking the users: by varying the total users to (a) $2^{10}$, (b) $2^{15}$, and (c) $2^{20}$. The header size is measured by the number of group elements, where a single group element is a 20-bytes object from the pairing-based cryptography (PBC) library. In all cases, the CSD-based broadcast encryption outputs the smallest headers, compared to the SD-based and interval-based broadcast encryption.

**Key Size.** The CSD-based broadcast encryption is also efficient in terms of key sizes; it maintains a public key and secret key size comparable to the standard broadcast encryption such as SD-based and interval-based broadcast encryption.

Table 3 shows the public key (PK) size and secret key (SK) size of the CSD-based, SD-based, and interval-based broadcast encryption. The public key size in CSD-based broadcast encryption is proportional to the user depth *n*, within the same order of O(logn) as the SD-based and interval-based construction. The secret key size is similar to the interval-based construction, within the same order of $O(\log^{2}n)$ for user depth *n*.

**Performance.** For the performance of encryption and decryption, the CSD-based broadcast encryption improves the encryption and decryption time compared to the SD-based and interval-based broadcast encryption. The main factor is that our CSD-based construction does not involve any public parameter related computation, while the other constructions require additional delegation from the public parameters which is proportional to the user depth.

Table 4 shows the encryption time and decryption time of the CSD-based, SD-based, and interval-based broadcast encryption, by varying the user depth from 10-bits to 20-bits (total users of $2^{10},2^{15},2^{20}$). Our CSD-based broadcast encryption maintains almost the same performance for all cases, while the other constructions suffer from the increase of encryption and decryption time proportional to the user depth.

**Open Source.** The experiment resources are publicly available at GitHub as an open source (https://github.com/snp-lab/CSD), for the open science and reproducible research. The resources consist of *subset construction* algorithm for each combinatorial subset difference (CSD), subset difference (SD), and interval, and *broadcast encryption* implementation for CSD-based, SD-based, and interval-based scheme.

## 10. Conclusions

We propose the combinatorial subset difference (CSD), which is a new subset representation for the broadcast encryption. The CSD representation is the most general representation compared to state-of-the-arts such as subset difference (SD) or interval representations. Due to the general coverage, the CSD representation generates minimal subsets which lead to the minimal header size when applied to the broadcast encryption. The CSD representation is the only representation to cover the non-hierarchical IP addresses, making it as a suitable choice for IoT network multicast. We design and implement the algorithm for CSD subset generation, and verify the practicality of our CSD in the IoT systems.

We also propose the CSD-based public key broadcast encryption, which can be efficiently applied to the IP multicast in IoT systems. The CSD-based broadcast encryption achieves minimal (total) header size due to the minimal subsets of the CSD representation, and it also improves the encryption time and decryption time compared to the existing broadcast encryption. Our experimental results verify that the CSD-based broadcast encryption reduces the header size 31% for the worst cases, improves encryption time by 6 times, and improves decryption time by 10 times compared to the SD-based broadcast encryption. For the security, we prove the collusion-resistance and semantic security of the CSD-based construction under the standard *l*-BDHE assumption, and extend the construction to the CCA-secure broadcast encryption.

## Figures and Tables

**Table 1:** Size and performance comparison among public key broadcast encryptions.

	Public Key SD ([6,7])	Interval [10]	Revocable SD [11]	CSD
**PK (size)**	logn	2logn	7×8	4logn
**SK (size)**	$\log^{3}n$	$2\log^{2}n$	$4\log^{2}n\times 8$	$3\log^{2}n$
**Hdr (size)**	4r	3r	(3×8+1)·2r	2r
**# subsets**	2r	*r*	2r	*r*
**# group elements**	2	3	3×8+1	2
**Enc (computation)**	2relogn	4re	12re	3re
**Dec (computation)**	elogn+2p	2elogn+4p	e+4p	2e+2p
**Assumption**	l−BDHI	l−BDHE	DBDH	l−BDHE

*e* = point multiplications, *p* = pairings, *n* = total users, *r* = revoked users.

**Table 2:** Type solution in SD and CSD algorithms.

$T_{0}$	$T_{1}$	Type in SD	Type in CSD	Subset Generation
⊥	⊥	⊥	⊥	0
⊥	⊤	*S*	*S*	1
⊥	*S*	⊥	⊥	0
⊤	⊤	⊤	⊤	0
⊤	*S*	*S*	*S*	0
*S*	*S*	⊥	*S*	0

**Table 3:** Comparison of key sizes in broadcast encryption.

	Depth	Public Key SD ([6,7])	Interval [10]	CSD (Ours)
PK (KB)	10-bit	0.25	0.49	0.83
	15-bit	0.37	0.73	1.24
	20-bit	0.50	0.98	1.65
SK (KB)	10-bit	19.61	4.03	5.92
	15-bit	66.18	9.07	13.32
	20-bit	156.88	16.13	23.69

**Table 4:** Comparison of encryption time and decryption time (per subset) in broadcast encryption.

	Depth	Public Key SD ([6,7])	Interval [10]	CSD (Ours)
Enc (s)	10-bit	0.70	0.24	0.20
	15-bit	0.94	0.25	0.20
	20-bit	1.18	0.25	0.20
Dec (s)	10-bit	1.02	1.67	0.17
	15-bit	1.37	2.41	0.17
	20-bit	1.75	3.17	0.17
